# Functional Parcellation of the Cerebral Cortex Across the Human Adult Lifespan

**DOI:** 10.1093/cercor/bhy218

**Published:** 2018-10-11

**Authors:** Liang Han, Neil K Savalia, Micaela Y Chan, Phillip F Agres, Anupama S Nair, Gagan S Wig

**Affiliations:** 1 Center for Vital Longevity and School of Behavioral and Brain Sciences, University of Texas at Dallas, Dallas, TX, USA; 2 Yale University School of Medicine, New Haven, CT, USA; 3 Department of Psychiatry, University of Texas Southwestern Medical Center, Dallas, TX, USA

**Keywords:** aging, functional connectivity, networks, parcellation, resting-state

## Abstract

Adult aging is associated with differences in structure, function, and connectivity of brain areas. Age-based brain comparisons have typically rested on the assumption that brain areas exhibit a similar spatial organization across age; we evaluate this hypothesis directly. Area parcellation methods that identify locations where resting-state functional correlations (RSFC) exhibit abrupt transitions (boundary-mapping) are used to define cortical areas in cohorts of individuals sampled across a large range of the human adult lifespan (20–93 years). Most of the strongest areal boundaries are spatially consistent across age. Differences in parcellation boundaries are largely explained by differences in cortical thickness and anatomical alignment in older relative to younger adults. Despite the parcellation similarities, age-specific parcellations exhibit better internal validity relative to a young-adult parcellation applied to older adults’ data, and age-specific parcels are better able to capture variability in task-evoked functional activity. Incorporating age-specific parcels as nodes in RSFC network analysis reveals that the spatial topography of the brain’s large-scale system organization is comparable throughout aging, but confirms that the segregation of systems declines with increasing age. These observations demonstrate that many features of areal organization are consistent across adulthood, and reveal sources of age-related brain variation that contribute to the differences.

Brain anatomy and function differ with age; these differences can be observed across the brain’s spatial scales, and from birth through older adulthood (Johnson 2001; Hedden and Gabrieli 2004). At a macroscopic level, the brain is organized into distinct cortical areas and subdivisions of subcortical structures (Sejnowski and Churchland 1989), a spatial organization revealed by the convergence of regional differences divided by local transitions in patterns of function, architectonics, connectivity and/or topography (Van Essen et al. 1981; Felleman and Van Essen 1991). Efforts to directly evaluate age-related differences in the anatomy and function of brain areas and subcortical structures have greatly benefited from the development of sophisticated image processing techniques. These methods typically align subjects to one-another, and to a common reference space using each individual’s neuroanatomical features (Fischl et al. 1999; Buckner et al. 2004; Van Essen et al. 2012). An underlying assumption across studies evaluating age-related differences in brain organization and function following anatomical alignment is that the spatial organization of functional areas remains relatively consistent across individuals and across age. However, while there is evidence that areal divisions are established early in development (for review see Gilmore et al. 2018), the boundaries of areas have also been shown to exhibit experience-dependent alterations in animal models (Seelke et al. 2016). A few additional points are also worth considering: 1) brain areas do not respect morphometric landmarks (Van Essen et al. 1981; for review see Wig et al. 2011); 2) in adulthood, increasing age is associated with increasing anatomical change and variability (Magnotta et al. 1999; Sowell et al. 2003; Raz et al. 2005); and 3) even among similarly aged individuals, there can be substantial variation in area localization (Caspers et al. 2006; Glasser et al. 2016). As such, it is important to evaluate whether and how the spatial arrangement of functional areas differs across age in order to accurately understand age-related changes in brain organization and function. Here, we perform this area parcellation evaluation, examining age-defined cohorts sampled from a broad segment of the adult lifespan (20–93 years).

How and why might adult aging be associated with differences in the spatial arrangement (topography) of functional areas? Healthy aging is accompanied by the progressive thinning of the cerebral cortex and reductions in volume and surface area, particularly in prefrontal, temporal, and parietal cortex (Resnick et al. 2003; Raz et al. 2005; Fjell et al. 2009; Storsve et al. 2014). These differences in macro-anatomy exhibit greater variability with increasing age (Sowell et al. 2003), thus, compromising anatomical alignment for older age groups (Fischl and Dale 2000; Good et al. 2001). As such, it is likely that area alignment may differ across individuals from different age segments even when their brains have been aligned to a common reference space. However, it is also important to acknowledge that the differences in gray-matter cortical thickness are caused by cellular changes (including shrinkage and loss of neurons (Kril et al. 2004), loss of dendritic spines of cells observed in both rats (Markham and Juraska 2002) and humans (Uylings and de Brabander 2002; for review see Burke and Barnes 2006)), and that these changes impact neural function (Barnes et al. 1983; Chang et al. 2005) and synaptic connectivity (Barnes and McNaughton 1980; for review see Morrison and Baxter 2012). Accordingly, it is possible that age-related changes in function and anatomy may result in additional functional distinctions within areas resulting in increased-partitioning of the area (or increased “differentiation”), or between areas at their transition zones resulting in decreased partitioning of areas (greater “blurriness” of boundaries).

Area parcellation has a long history and has largely required invasive measurement of brain features including direct neural recording (Penfield and Jasper 1954), cortical stimulation (Penfield and Boldrey 1937; Lee et al. 2000), and postmortem analysis of anatomy (Brodmann 1909; Hof et al. 1995; Öngür et al. 2003; Scheperjans et al. 2008). Advances in the acquisition and analysis of noninvasive imaging have provided methods to differentiate area function and organization with greater ease (Petersen et al. 1988; Johansen-Berg et al. 2004; Amunts and Zilles 2015). More recently, parcellation procedures have been developed that identify locations where patterns of resting-state functional correlations (RSFC) (Biswal et al. 1995) exhibit abrupt transitions (i.e., putative areal boundaries) (Cohen et al. 2008; Wig, Laumann and Petersen 2014; Laumann et al. 2015; Gordon et al. 2016; Xu et al. 2016). Boundary mapping of resting-state signals has been shown to exhibit correspondence with areal divisions that have been defined by patterns of cyto- and myelo-architectonics (Wig, Laumann and Petersen 2014; Glasser et al. 2016; Gordon et al. 2016) and functional activity (Wig, Laumann and Petersen 2014; Laumann et al. 2015). Importantly, the procedures can be rapidly deployed across the entire cerebral cortex without the need of dissociating task-related activation patterns. To date, resting-state boundary mapping techniques have predominantly been applied to young adult brains. It remains unclear whether and where area boundaries differ with increasing age; the application of these methods towards parcellating the brains of groups of individuals from across the adult lifespan provides an unprecedented opportunity to examine whether the RSFC-defined cortical organization of brain areas differs with increasing age.

Here, we utilize and refine RSFC-based boundary mapping methods (Wig, Laumann and Petersen 2014; Gordon et al. 2016) to create functional parcellations for 5 independent age cohorts that were evenly sampled across the adult lifespan (20–93 years; Table 1). The parcellations across the 5 cohorts are evaluated and validated in a series of tests that probe RSFC consistency in parcels using 2 separate datasets. While the majority of the prominent features of area parcellation are consistent across the adult lifespan, differences in area parcellation exist and are more pronounced with increasing age. Our analyses reveal that many of these differences are mediated by age-related differences in brain structure and anatomical alignment. We demonstrate how cohort-specific parcellations serve as more accurate representations to understand patterns of task-related activity and functional connectivity. Importantly, we apply these parcellations to analyses of large-scale networks across age. Using cohort-specific parcellations to define cohort-specific brain network nodes reveals that the spatial organization of large-scale resting-state brain networks is largely unaltered with increasing age, while the previously reported differences in network organization across age (system segregation; Chan et al. 2014; Wig 2017) are still evident when using nodes defined from cohort-specific parcellation.

**Table 1:** Participant demographics. Sex distribution was compared across cohorts using $\chi^{2}$ tests; MMSE scores were compared using *F* tests (*F*_Dataset1(4, 131)_ = 4.805, *P* < 0.001; *F*_Dataset2(4, 217)_ = 9.318, *P* < 0.001). MMSE, Mini-Mental State Examination; NA, not available; ns, not significant

Cohorts	Age range	*N*		Female (%)		MMSE score	
		Dataset 1	Dataset 2	Dataset 1	Dataset 2	Dataset 1	Dataset 2
Younger adults (YA)	20–34 y	26	62	69	60	29.48 (0.77)	28.66 (1.19)
Middle early adults (ME)	35–49 y	29	49	69	65	29.45 (0.95)	28.77 (1.12)
Middle late adults (ML)	50–64 y	34	43	71	67	29.56 (0.66)	28.42 (1.14)
Older early adults (OE)	65–79 y	32	38	66	58	28.91 (1.30)	27.84 (1.22)
Older late adults (OL)	80–93 y	16	30	81	57	28.44 (1.41)	27.43 (1.10)
*P*	NA	NA	NA	ns	ns	<0.001	<0.001

## Materials and Methods

### Participants

Two separate datasets were used. Initially, these datasets were analyzed separately: Dataset 1 was used to generate cohort-specific parcellations, and Dataset 2 served as a separate replication sample for purposes of evaluating parcellation quality. In the final analysis, the datasets were combined to generate cohort-specific parcellation maps using all of the available data. Participants were recruited from the Dallas–Fort Worth community and provided written consent before participating. All study procedures were reviewed and approved by the Institutional Review Boards at The University of Texas at Dallas and The University of Texas Southwestern Medical Center. All subjects were neurologically normal, right-handed, native English speaking healthy adults from the Dallas Lifespan Brain Study (DLBS). In the study recruitment, individuals were excluded due to 1) chemotherapy in the past 5 years, 2) coronary bypass surgeries, 3) major substance abuse, 4) disorders of the immune system, 5) loss of consciousness for more than 10 min, 6) Mini-Mental State Examination (MMSE) scores less than 26, and 7) any MRI safety contraindications.

The cohorts defined by the 2 datasets were different in several respects, including the amount of data per subject, subjects constituting each cohort, and cohort sample size. While Dataset 2 contains more subjects, there is less data available per subject (see Imaging Data Acquisition). As RSFC parcellation quality relates to the amount of available data (Laumann et al. 2015), the dataset with less subjects but more data per subject (Dataset 1) was used for cohort-specific parcellation generation and this second dataset (Dataset 2) was used for cross-sectional validation on the parcellation results from Dataset 1.

(1) Dataset 1 comprised of data from 206 subjects (age range: 24–93 y). Those with high quality data (see RSFC Preprocessing) were included in the final sample (*N* = 137) and categorized into 5 age cohorts based on their age at the time of data collection (i.e., younger adults [YA], middle early adults [ME], middle late adults [ML], older early adults [OE], older late adults [OL]; for the age ranges of the cohorts, see Table 1).

(2) Dataset 2 included data collected from 222 subjects (age range: 20–89 y; Table 1). The subjects in this dataset include both the subjects in Dataset 1 (Dataset 2 was collected roughly 3.5 years prior to the collection of Dataset 1) and a number of additional subjects. Subjects from Dataset 2 were categorized into 5 cohorts based on their age at time of data collection. In total there were 105 subjects overlapping across Datasets 1 and 2 (percentage of overlapping subjects in Dataset 2: YA = 43%, ME = 39%, ML = 51%, OE = 63%, OL = 47%).

### Imaging Data Acquisition

Brain images were acquired with a Philips Achieva 3 T whole-body scanner (Philips Medical Systems, Bothell, WA) and a Philips 8-channel head coil at the University of Texas Southwestern Medical Center using the Philips SENSE parallel acquisition technique.

#### Anatomical Images

A T1-weighted sagittal magnetization-prepared rapid acquisition gradient echo (MP-RAGE) structural image was obtained (TR = 8.1 ms, TE = 3.7 ms, flip-angle = 12°, FOV = 204 × 256 mm^2^, 160 slices with 1 × 1 × 1 mm^3^ voxels).

#### Functional Images

Functional magnetic resonance imaging (fMRI) used a blood oxygenation level-dependent (BOLD) contrast sensitive gradient echo echo-planar sequence (TR = 2000 ms, TE = 25 ms, flip angle = 80°, FOV = 220 mm, 43 interleaved axial slices per volume, 3.5/0 mm (slice-thickness/gap), in-plane resolution = 3.4 × 3.4 mm^2^). At the beginning of each run, 5 volumes were acquired and discarded to allow the MR signal to reach a steady-state.

- *Resting-state fMRI*: There were 2 functional runs in Dataset 1, and 1 functional run in Dataset 2. Each functional run consisted of 180 BOLD acquisitions for Dataset 1 and 154 acquisitions for Dataset 2. Subjects were instructed to remain still while fixating on a white crosshair against a black background. The experimenter verified that subjects complied with the instructions and did not fall asleep during the functional scan via verbal confirmation.
- *Task-evoked fMRI*: Task-evoked data from 2 types of tasks were used in the current study (collected in the same study session as RSFC data from Dataset 2; for additional details see Chan et al. 2017): 1) Word judgment scans (semantic task) collected in a block design (one run, 231 volumes; *n* = 222) where subjects viewed 128 words and made living or nonliving judgments. Stimuli were categorized into 2 conditions (unambiguously living or nonliving [Easy condition] vs. ambiguous and harder to classify as living or nonliving [Hard condition]). The 2 conditions were modeled separately for the analysis in this study. All stimuli were presented in lowercase white font at the center of a black screen. 2) Scene classification scans (visual task) collected in an event-related design (3 runs, 171 volumes in each; *n* = 218) in which subjects viewed colored images of outdoor landscapes and determined whether there was water present in the scene. Based on a subsequent memory test, stimuli were categorized into 2 conditions (successfully remembered [Remember condition] vs. forgotten [Forget condition]) and were modeled accordingly. All picture stimuli were presented at the center of a black screen.

### Basic fMRI Preprocessing

A number of steps were taken to reduce artifacts in functional images, including 1) correction of odd versus even slice intensity differences attributable to interleaved acquisition without gaps, 2) head movement correction within and across runs, and 3) across-run intensity normalization to a whole brain mode value of 1000 (Miezin et al. 2000).

### RSFC Preprocessing

Additional preprocessing steps were taken to reduce spurious variance unlikely to reflect neuronal activity in RSFC data (Power et al. 2014). 1) Multiple regression of the BOLD data to remove variance related to the whole brain gray matter signal (global signal; defined by each subject’s own anatomy), ventricular signal, white matter signal, 6 detrended head realignment parameters obtained by rigid-body head motion correction, and the first-order derivative terms for all aforementioned nuisance variables. Global signals have been shown to be coupled with neural activity (Schölvinck et al. 2010), but recent evidence has revealed that a major component of the global signal consists of spatially nonspecific signal artifacts, in which head motion can play a significant role (Satterthwaite et al. 2013, 2013; Power et al. 2014, 2017). Because older adults are more prone to head movement (Van Dijk et al. 2012; Savalia et al. 2017) that leads to altered RSFC profiles, it is critical to minimize the source of bias that may contribute to erroneous estimation of RSFC areal boundaries. 2) Band-pass filtering (0.009 Hz < *f* < 0.08 Hz).

To further reduce the effect of motion artifact on RSFC, data were processed using a “scrubbing” procedure (Power et al. 2014). Motion-contaminated volumes were first identified by their frame-by-frame displacement (FD) calculated as the sum of absolute values of the differentials of the 3 translational motion parameters and 3 rotational motion parameters (Power et al. 2014). Volumes with FD > 0.3 mm were flagged. In addition, the frames acquired immediately prior and immediately after each of these frames were also flagged to account for temporal spread of artifactual signal resulting from the temporal filtering in the first RSFC preprocessing iteration.

The RSFC preprocessing steps outlined above (steps 1 and 2; including nuisance regression and temporal filtering) were applied in the second iteration on RSFC data that excluded volumes flagged during motion scrubbing. Following data scrubbing, any participant with less than 75 frames of remaining data were removed from subsequent analyses, resulting in 137 subjects (Dataset 1) and 222 subjects (Dataset 2). In Dataset 1, 254 frames were retained for each subject on average across all cohorts (range: 80–403). In Dataset 2, each subject on average retained 140 frames across all cohorts after scrubbing (range: 75–154).

### Processing of Cortical Surface and Subcortical Anatomy

Each subject’s cortical surface was reconstructed to map functional data, analyze and visualize data in a standard surface space, and to rule out the possible contribution of white matter signal by sampling signals from the gray ordinate to the surface (partial volume effects). The anatomical volumes in their “native” space were first submitted to FreeSurfer v5.3 automated processing pipeline. (Dale et al. 1999; Fischl et al. 1999; Ségonne et al. 2005). This pipeline includes brain extraction, cortical and subcortical segmentation, generation of the gray matter-white matter boundary (white matter surface) and outer cortical surface (pial surface), inflation of the surfaces to a sphere, and surface shape-based spherical registration of the subject’s “native” surface to the fs-average surface.

We recently demonstrated that excessive head motion in structural images can lead to inflated effect sizes in the relationships between regional anatomical measures and age (Savalia et al. 2017). In addition, the gray/white matter signal intensity ratio of T1-weighted image increases with aging (Salat et al. 2009), which may systematically bias estimation of specific subjects’ brain data as anatomical processing algorithms are sensitive to these contrasts (Dale et al. 1999). Accordingly, additional steps were taken to reduce the influence of head motion and other possible aging-related confounds. The automated FreeSurfer outputs for each subject were manually inspected and edited as necessary and verified by an independent researcher. Manual editing included removal of nonbrain tissue misclassified as part of the cortical surface, and editing regional voxel intensity values of tissue excluded from the white and pial surfaces (misclassification of gray and white matter). The manual editing is particularly important to the current study involving aging data, which are more susceptible to age-related anatomical abnormalities possibly mishandled by the default FreeSurfer pipeline (Savalia et al. 2017). The editing follows instructions from the official FreeSurfer Wiki and editing tutorials (http://freesurfer.net/fswiki/FreeSurferWiki; https://surfer.nmr.mgh.harvard.edu/fswiki/FreeSurferBeginnersGuide), and an in-house guide to our laboratory’s FreeSurfer editing procedures (available for download at our laboratory’s website). T1-weighted image quality control (QC) ratings by 2 researchers (N.S. and P.A.) and in-scanner head motion for each functional run that was quantified with FD (Power et al. 2014) were then used to flag subjects with excessive motion (see Savalia et al. 2017 for detailed procedures), who were excluded from the analyses probing the relationship between cortical thickness difference and parcellation overlapping percentages.

A single deformation map was created for each subject by combining 1) the deformation map from “native” space to FreeSurfer’s fsaverage atlas and 2) the deformation map from fsaverage-aligned data to a hybrid left-right fsaverage surface (fs_LR; Van Essen et al. 2012). The individuals’ surfaces in “native” space were then registered to the 164k fs_LR atlas using this single deformation map in a one-step resampling procedure and down-sampled to 32k standard mesh (Van Essen et al. 2012).

Due to age-related volume shrinkage of subcortical gray matter (Pfefferbaum et al. 1994; Good et al. 2001; Fox and Schott 2004) and individual variability in change across age (Raz et al. 2005), the subcortical structures and cerebellum were aligned across subjects (across all cohorts) to provide more precise comparison of anatomical features between subjects and to better align functional data. The volumetric subcortical structures and cerebellum labeled by FreeSurfer in “native” space were registered to the DLBS adult-lifespan atlas (Chan et al. 2014) to reduce greater registration error among older adults caused by conventional registration to younger adults-derived MNI atlas space (Buckner et al. 2004). Atlas transformation was computed for each subject using the atlas-registered anatomical image.

### CIFTI File Generation

The files of Connectivity Informatics Technology Initiative (CIFTI; Glasser et al. 2013) format were generated to integrate information from all possible brain ordinates (such as the cerebral cortex, subcortical structures, and the cerebellum). To do so, the time series data of the cortical surface were derived by resampling functional volumes in “native” space to 32k mesh surface using single deformation maps derived from surface data processing and smoothed on the surface with a Gaussian kernel (6 mm full width-half maximum [FWHM]). Volumetric time series of each individual’s subcortical structures and cerebellum were created by resampling the functional data to an isotropic 3 mm atlas space, combining movement correction and atlas transformation in a single cubic spline interpolation (Lancaster et al. 1995; Snyder 1996). This single interpolation procedure eliminated blurring that would be introduced by multiple interpolations. Finally, the subcortical data were smoothed in volumetric space with a Gaussian kernel (6 mm FWHM).

Finally, the time series data of the cortical surface and volumetric time series of FreeSurfer-labeled subcortical structures and the cerebellum were combined to create the CIFTI files.

### Task-Evoked fMRI Analysis

Task-evoked fMRI task data were analyzed using SPM 8 (Wellcome Department of Cognitive Neurology; http://www.fil.ion.ucl.ac.uk/spm/). For each task condition, a general linear model (GLM) was built to include the onsets and durations of each block/event and the number of runs where appropriate, and nuisance regressors (the 6 head realignment parameters, a high-pass filter and linear trends) for each subject. Following a subject’s GLM estimation, the volumetric task-evoked beta image was mapped to the 32k fs_LR atlas surface using single deformation maps derived during surface data processing.

### Generation of Parcellation Maps

A flowchart of the parcellation map generation procedure for each cohort can be seen in Figure S1. For each subject, the pairwise correlation of the time series between each surface vertex and every other surface vertex in the CIFTI file was computed to create a 32 492 × 64 984 (32k × 64k) correlation matrix for each hemisphere (similar to the procedure described in previous work) (Wig, Laumann, Cohen, et al. 2014; Gordon et al. 2016). Because the number of subcortical voxels varied across subjects, their signals were not included to create cortical parcellations, which relied on equivalent matrix sizes across participants in order to create bootstrap samples (described below). Each correlation map was transformed using Fisher’s *r*-to-*z* transformation; the full correlation distribution (including negative and positive correlations) was used for the generation of parcellation maps.

To generate reliable parcellation maps that both reduce the degree of individual variability and also minimize sampling error of RSFC profiles that may be introduced with inclusion of lesser time points per subject (Laumann et al. 2015), a bootstrapping procedure was used for parcellation map generation. 100 bootstrap samples were created for each cohort, where each bootstrap sample was created by randomly sampling (with replacement) the same number of subjects for the cohort. For each bootstrap sample in a given cohort, the following steps were then taken: 1) Mean correlation maps were generated by averaging correlation maps across the subjects in the bootstrap sample, producing a 32k × 64k matrix for each hemisphere. 2) Vertex-wise RSFC similarity matrices were derived by calculating the pairwise spatial correlations between every vertex’s RSFC correlation maps with one another, resulting in a 32 492 × 32 492 (32k × 32k) similarity matrix (for each hemisphere). Each slice of the similarity matrix represents a map on the cortical surface in which values represent the similarity between RSFC maps of a given vertex and all other vertices. 3) To identify positions where RSFC similarity exhibited abrupt transitions, gradient maps were generated by computing the first spatial derivative of RSFC similarity maps using the “cifti-gradient” function in the Connectome Workbench (Glasser et al. 2013). This resulted in a 32k × 32k gradient matrix (for each hemisphere). 4) Each of the resultant gradient maps were smoothed with a Gaussian kernel (6 mm FWHM) on the surface (as in Gordon et al. 2016).

Each cohort’s average gradient map was calculated by averaging the gradient maps across the 100 bootstrap samples to produce a 32k × 32k matrix. For each cohort, a watershed algorithm (Beucher and Lantuéjoul 1979) was applied to each slice of this average gradient matrix to identify cortical boundaries, creating a 32k × 32k boundary map matrix. Here, each column represents a given vertex’s watershed boundary image whereby each value in the boundary matrix (**A**_ij_) represents whether the *i*th vertex is a boundary (**A**_ij_ = 1) or not (**A**_ij_ = 0) in the *j*th vertex’s watershed boundary map. Lastly, each cohort’s final boundary map (32k × 1 per hemisphere) was created by averaging across all vertices’ boundary maps.

Closely following Gordon et al. (2016), the functional parcellation of each age cohort was derived from its final boundary map. We first identified local minima of each cohort’s boundary map that were then used to detect parcels using a watershed algorithm. This procedure required thresholding boundary values when generating parcels, which poses a limitation to the parcellations generated using this approach. To avoid arbitrarily choosing a threshold, we explored a wide range of thresholds (5–60%) to assess the results and compare them to the boundary maps. Due to the nature of the watershed algorithm, parcellations at lower thresholds often appeared over-segmented, and were under-segmented at higher thresholds because the algorithm neglected weaker borders that may delineate important RSFC features. Based on visual examination and comparison between these parcels and those from existing studies (Glasser et al. 2016; Gordon et al. 2016), we used the parcellations at the threshold of 35%, which not only captured important boundaries while avoiding over- and under-segmentation across cohorts, but also shared more features in common with the existing parcellation schemes relative to parcellations obtained from other thresholds.

### Comparison and Evaluation of Boundary Maps and Parcellations

#### Comparison of Boundary Maps

The similarity of boundary maps between YA and each of the non-YA cohorts (ME, ML, OE, OL) was quantified using Dice coefficients. To further investigate whether the similarity between maps was better than chance, the Dice coefficients between each map comparison was compared with a corresponding null model. The null model was derived by calculating the Dice coefficients between the boundary map of each non-YA cohort and 100 randomly rotated YA boundary maps on the spherical 32k fs_LR atlas (Gordon et al. 2016). Vertices falling in the medial wall area of the rotated boundary map were assigned with the mean boundary value of the same vertices in all other valid rotated boundary maps where the vertex did not fall in the medial wall. A *Z*-score $\left(Z_{boundary\;maps}\right)$ was computed using the coefficients with the formula below:

$$Z_{boundary\;maps}\;=\;\frac{DiceCoef_{actual}-\;M_{DiceCoef_{null}}}{SD_{DiceCoef_{null}}}$$

where $DiceCoef_{actual}$ is the Dice coefficient of YA’s boundary map with a given non-YA boundary map, $M_{DiceCoef_{null}}$ is the mean of 100 null model Dice coefficients, and $SD_{DiceCoef_{null}}$ represents the standard deviation (SD) of 100 null model Dice coefficients. Dice coefficients and *Z*_boundary maps_ were computed based on the strongest boundaries (i.e., higher RSFC transition probability) across multiple thresholds (top 1-100%, in steps of 1%).

#### Evaluation of Parcellation Quality Using Homogeneity Tests

The parcellation of each cohort was evaluated with homogeneity tests performed as follows (Gordon et al. 2016): Firstly, for a given vertex in a parcel, whole-brain connectivity patterns (i.e., cortical RSFC maps) were averaged across all subjects. A principal component analysis (PCA) was performed on the averaged connectivity patterns of all vertices for a given parcel, where the variance explained by the first component represents the homogeneity value for that parcel. The homogeneity values of all parcels were then averaged to produce the mean homogeneity for each cohort’s parcellation. To inspect whether this parcellation homogeneity was better than chance, we calculated the *Z*-scores $\left(Z_{homogeneity}\right)$ on both the mean homogeneity of each cohort’s actual parcellation and a null model comprising mean homogeneities from 100 randomly rotated parcellations for this cohort. Any parcels rotated into the medial wall area when generating the null model parcellations had their homogeneity values assigned as the mean homogeneity value of the same parcel from all other null model rotations in which the parcel did not fall into the medial wall.

To determine whether the application of YA parcellation to non-YA cohort data also exhibited homogenous parcel estimates, we performed similar homogeneity tests as described above, but this time using the YA parcellation applied to each non-YA cohort data. For each non-YA cohort, a *Z*-score $\left(Z_{homogeneity}\right)$ was computed based on the mean homogeneity using YA parcellation applied to the cohort’s data and a null model comprising mean homogeneities from 100 randomly rotated YA parcels. A comparison was made between the *Z*-scores, instead of mean homogeneities, from cohort-specific and YA parcellations to avoid the issue of parcel sizes affecting homogeneity measures.

For each dataset, both the cohort-specific and YA parcellations (generated from Dataset 1) were applied to each cohort’s data and $Z_{homogeneity}$ values were computed and compared for each type of parcellation.

#### Evaluation of Parcellation Quality Using Silhouette Coefficient

The Silhouette coefficient (Rousseeuw 1987) describes the reliability of assigning vertices to their parcels (Yeo et al. 2011; Craddock et al. 2012; Arslan et al. 2018):

$$\mathrm{Silhouette}\;\mathrm{coefficient}=\;\frac{b_{i}-\;a_{i}}{Max\left(b_{i},a_{i}\right)}$$

where $b_{i}$ is the mean of dissimilarity values (1 – correlation coefficients) between the whole-brain RSFC map of vertex *i* and the whole-brain RSFC maps of all the vertices in the immediately adjacent parcels, and $a_{i}$ denotes the mean of dissimilarity values between the whole-brain RSFC map of vertex *i* and the whole-brain RSFC maps of all other vertices in the same parcel. Silhouette coefficient ranges from −1 to 1 wherein a positive value suggests the RSFC map of a given vertex is more similar to all other vertices in the same parcel relative to the vertices in the immediately adjacent parcels.

For each dataset, both the cohort-specific and YA parcellations (generated from Dataset 1) were applied to each cohort’s data and Silhouette coefficient values were computed for each type of parcellation. Silhouette coefficient values using cohort-specific and YA parcellations were compared using a 2-sample *t*-test.

#### Comparison of Spatial Similarity of Parcellations Across Cohorts

Two techniques were used to assess the spatial similarity of the YA cohort parcellation with each non-YA cohort's parcellation:

- *Matching parcels*: Parcels between each non-YA cohort and YA were matched based on the spatial overlapping percentages computed using Jaccard index. The formula of overlapping percentage was calculated as follows:
  $$Overlap=\;\frac{\left|P_{A_{i}}\cap^{\;}P_{B_{j}}\right|}{\left|P_{A_{i}}\cup^{\;}P_{B_{j}}\right|}$$
  where |⋅| denotes the number of objects (vertices) in a set, $P_{A_{i}}$ represents the *i*th parcel of cohort *A* and $P_{B_{j}}$ is the *j*th parcel of cohort *B*. This formula takes spatial features including shape, size, and location of parcels into account to provide an objective measure of the spatial similarity between 2 parcels across distinct cohorts. Parcels were matched with one another based on their maximal overlap across cohorts. This criterion can result in multiple matches for a given parcel. The average parcel overlap was calculated for each cohort relative to one another.
  Parcel matching across cohorts was evaluated in comparison to a null model. The null model comprised 100 overlapping percentages of matched parcellation pairs, in which each non-YA cohort’s parcellation was matched to each of 100 randomly rotated YA parcellations. A *Z*-score of overlapping percentages $\left(Z_{overlap}\right)$ was computed to quantify the matching results in comparison to the null model (for parcels matching to the rotated medial wall, the averaged overlapping percentage of its successfully matched parcel pairs was used):
  $$Z_{overlap}=\;\frac{M_{overlap_{actual}}-M_{overlap_{null}}}{SD_{overlap_{null}}}$$
  where $M_{overlap_{actual}}$ is the mean of overlapping percentages of all matched parcels in each cohort-pair, $M_{overlap_{null}}$ is the mean of the null model, and $SD_{overlap_{null}}$ represents the standard deviation of the null model.
  Spatially matched parcels were also compared in terms of the similarity of their RSFC using the combined dataset (the results were qualitatively similar when analyzed within each dataset). To do so, for each subject in a given cohort, the parcellation of this cohort was applied to the subject’s vertex-wise RSFC time series data to extract mean time series for each parcel. The mean time series of each parcel was then correlated with the time series of all cortical vertices to generate a RSFC map for each parcel in each subject. Finally, the RSFC map for each parcel was averaged across all subjects in the cohort. The RSFC similarity value of any matched parcels between 2 cohorts was calculated by correlating their cohort averaged RSFC maps. The RSFC similarity was quantified in comparison to a null model, which was generated as described above, using the formula (for parcels matching to the rotated medial wall in some null model matched parcellations, the averaged RSFC similarity of its successfully matched parcel pairs was used):
  $$Z_{RSFC}=\;\frac{M_{RSFC_{actual}}-M_{RSFC_{null}}}{SD_{RSFC_{null}}}$$
  where $M_{RSFC_{actual}}$ is the mean of RSFC similarity values of all matched parcels in 2 actual parcellations, $M_{RSFC_{null}}$ is the mean of the null model, and $SD_{RSFC_{null}}$ represents the standard deviation of the null model.
- *Adjusted rand index (ARI)*: The spatial similarity of 2 parcellations was quantified using the ARI (Hubert and Arabie 1985), a measure that represents a count of the vertices that are assigned to the same or different parcels (Thirion et al. 2014; Arslan et al. 2018).

A *Z*-score $\left(Z_{ARI}\right)$ was calculated based on ARI of actual parcellations between each non-YA cohort and YA in comparison to a null model. The null model comprised 100 ARI between each non-YA cohort’s actual parcels and 100 randomly rotated YA parcels:
  $$Z_{ARI}\;=\;\frac{ARI_{actual}-\;M_{ARI_{null}}}{SD_{ARI_{null}}}$$
  where $ARI_{actual}$ is the ARI between 2 actual parcellations, $M_{ARI_{null}}$ denotes the mean ARI of the null model, and $SD_{ARI_{null}}$ is the standard deviation of the null model.

### Measurement of Brain Structure and Anatomical Alignment

To evaluate whether specific factors may relate to between-cohort parcellation differences, the spatial distribution of differences in cortical thickness and anatomical alignment was quantified across cohorts. All available cortical thickness and anatomical deformation data from both Datasets 1 and 2 were used, although the results were qualitatively similar when analyzed within each dataset. Data from subjects identified as having excessive head motion (see Processing of Cortical Surface and Subcortical Anatomy for details) were excluded from the analysis.

#### Cortical Gray Matter Thickness

Each subject’s cortical gray matter thickness was calculated in FreeSurfer by computing the distance between the white matter surface and pial surface at each vertex along the cortical mantle. The resulting “native” space vertex-wise thickness map was then registered to the 164k fs_LR atlas using a single deformation map in a one-step resampling procedure. Individual vertex-wise thickness maps were averaged across subjects to create each cohort’s cortical thickness maps, producing a 164k × 1 vector (per hemisphere) wherein each value represents the average cortical gray matter thickness of each cohort at a given vertex. The cohort-average maps were down-sampled from 164k fs_LR atlas to 32k fs_LR atlas using Connectome Workbench (Glasser et al. 2013) so that the resultant images were in the same resolution as anatomical deformation maps and boundary maps.

#### Anatomical Deformation

The deformation map quantifies the data resampling density at each surface vertex from individual native space to atlas space, so it was used to approximate the degree of anatomical realignment due to variability of anatomy relative to the surface atlas. Each individual’s anatomical deformation map corresponds to the transformation between their native space surface mesh and the atlas-registered surface mesh, which was then resampled to 32k fs_LR atlas surface, resulting in a 32k × 1 vector (per hemisphere). Within each cohort, the individual maps were averaged across subjects to create a mean cohort deformation map.

### Agreement of Parcellations With Task-Evoked fMRI Maps

The quality of cohort-specific parcellations was evaluated based on the degree to which the parcels captured task-evoked estimates. We hypothesized that cohort-specific parcellations should be able to capture more uniform signals in task-evoked estimates within each parcel, in comparison to applying parcels defined from younger adults to cohorts of other ages. For each cohort, the corresponding cohort-specific parcellation was first applied to the cohort’s mean beta map for each task condition. Next, the SD of beta values within each parcel was computed and averaged across conditions of each task. The mean SD across all parcels was computed to summarize the general agreement of the parcellation with beta maps of each task. A *Z*-score $\left(Z_{SD}\right)$ was computed based on the averaged SD of each cohort-specific parcellation and a null model of 100 averaged SD values from applying 100 random rotations of the cohort-specific parcellation to the beta map, using the following formula (any parcel rotated into the medial wall in some null model rotations was assigned with the averaged SD of the same parcel that did not fall into the medial wall; the YA parcellation applied to each non-YA cohort’s beta map was evaluated using the same method):

$$Z_{SD}=\;\frac{M_{SD_{actual}}-M_{SD_{null}}}{SD_{SD_{null}}}$$

where $M_{SD_{actual}}$ denotes the mean of SD values across actual parcels, $M_{SD_{null}}$ is the mean of the null model, and $SD_{SD_{null}}$ is the standard deviation of the null model.

### Brain Network Analysis

Individual RSFC data in Dataset 2 were used in brain network analysis in order to confirm our previously reported findings (Chan et al. 2014) within the same dataset, and also avoid introducing potential variance related to within-subject longitudinal changes of brain networks that would accompany inclusion of both datasets.

#### Construction of Brain Graphs

Each subject’s brain graph was constructed using network nodes that were defined according to the individual’s cohort-specific parcels. The average time series across all vertices within each parcel was computed and cross-correlated with the average time series for every other parcel, producing a node-to-node correlation matrix for each individual. A Fisher’s *z*-transformed *r*-matrix (*z*-matrix) was then derived by converting correlation coefficients into *z*-values using Fisher’s *r*-to-*z* transformation. Although negative edges may represent meaningful network features (Rubinov and Sporns 2011), they were excluded from further analyses owing to the ambiguity in interpreting the negative correlations introduced by the necessary step of global signal regression (Power et al. 2017) and in accord with our previous work (Chan et al. 2014, 2017, 2018).

#### Community Detection and Comparison

Network communities of the node-wise graphs of all cohorts were identified using the Infomap algorithm (Rosvall and Bergstrom 2008). Specifically, a bootstrap approach was used to detect each cohort’s RSFC communities with the following steps: 1) 1000 bootstrap samples, each of which randomly sampled (with replacement) the same number of subjects from within the cohort, were created for each cohort. 2) The *z*-matrices were averaged across all subjects for each bootstrap sample and thresholded across 3–10% edge densities (in steps of 0.1% from 3% to 5% and in steps of 1% from 5% to 10%). This resulted in 1000 matrices at each edge density for each cohort. Correlations between nodes whose centroids were less than 20 mm apart were excluded from further analyses. 3) Community detection was performed on each thresholded mean *z*-matrix, resulting in 1000 community assignments for each cohort, at each edge density. The community assignments were labeled based on maximal overlap with a set of published RSFC functional systems (Power et al. 2011). 4) At each edge density, the most common assignment (mode) across the 1000 community assignments was selected as the node’s reliable community assignment.

To create the final community assignment for each cohort, a “consensus” procedure was used to collapse communities at each edge density. To do so, the system assignments of nodes at the sparsest edge densities were used, at which finer structures of the brain network could be revealed. Communities with 5 or fewer nodes (i.e., parcels) were excluded and their nodes were reassigned to the communities with 6 or more nodes at less sparse densities. Communities only present at one threshold were excluded. For a few nodes that belonged to major systems across most edge densities but were separated into small communities present only when connections became very sparse, we manually reassigned these nodes to the major systems from which they originated. For example, several parcels in the right lateral prefrontal cortex were predominantly assigned to the default mode community across most thresholds in ML, and a few parcels in the left lateral prefrontal cortex were predominantly assigned to the frontal–parietal community across most thresholds in OL. In the sparsest graph-density thresholds, these parcels were separated from the system they were assigned to across most densities and became isolated into independent communities. Since this was more of an over-partitioning effect due to the loss of edges in highly sparse matrices rather than the reflection of the whole assignment history across thresholds, we manually reassigned these parcels to the networks from which they originated. The unaltered assignments are presented in Figure S8. We note that summarizing community detection based on a consensus approach does not provide precise descriptions of every detected detail across edge densities, but rather a summary of general structure of the network across graph densities.

The spatial similarity of each system between each non-YA and YA cohort was quantified. Specifically, for each system, we calculated the Dice coefficient of the system maps between each non-YA and YA cohort $\left(DiceCoef_{actual}\right)$. To generate a null model, we created 100 random rotations of YA system maps, and then calculated 100 Dice coefficients (DiceCoef_null_) between each non-YA cohort system map and the randomly rotated system maps. Any non-YA system map falling within the medial wall of the randomly rotated YA system was assigned the mean value of all other null model Dice coefficients (where non-YA system did not fall into the medial wall). Finally, $Z_{system}$ was computed using the coefficients with the formula below.

$$Z_{system}\;=\;\frac{DiceCoef_{actual}-\;M_{DiceCoef_{null}}}{SD_{DiceCoef_{null}}}$$

where $M_{DiceCoef_{null}}$ is the mean of 100 $DiceCoef_{null}$ and $SD_{DiceCoef_{null}}$ represents the SD of 100 $DiceCoef_{null}$. As the mouth somatomotor system did not appear in 3 cohorts but was originated from the hand somatomotor system at less sparse edge density, we combined the 2 somatomotor systems together for this analysis.

#### System Segregation

A measure of brain system segregation was computed to summarize values of within-system correlations in relation to between-system correlations (Chan et al. 2014). Without thresholding the correlation coefficients, this measure takes the differences in mean within-system and mean between-system correlation as a proportion of mean within-system correlation, as noted in the following formula:

$$System\;Segregation=\frac{\overset{¯}{Z_{w}}-\overset{¯}{Z_{b}}}{\overset{¯}{Z_{w}}}$$

where $\overset{¯}{Z_{w}}$ represents mean connectivity (Fisher *z*-transformed correlation coefficients) between nodes within the same system and $\overset{¯}{Z_{b}}$ denotes mean connectivity between nodes of different systems. Based on the derived community assignments, system segregation was computed across all systems. In addition to the network’s overall system segregation summarizing connectivity across the entire brain network, system segregation was also calculated for two different types of functional systems (sensory-motor systems and association systems). Sensory-motor systems are primarily involved in processing sensory information (e.g., the visual system) and motor information (e.g., the hand somato-motor system), whereas association systems primarily integrate information across a wide range of tasks (e.g., cingulo-opercular control system). The system segregation of a given system type (i.e., association systems or sensory-motor systems) describes the extent to which systems within a given system-type (e.g., default mode, frontal–parietal, and others categorized as association systems) are segregated from all other functional systems. For more specific system type to system type segregation, such as association-to-association system segregation, $Z_{w}$ is the mean within-system connectivity of each association system, and $Z_{b}$ is the mean between-system connectivity of each association system to all other association systems. For the system segregation between different system types (e.g., association-to-sensory system segregation), $Z_{w}$ is the mean within-system connectivity of each association system, and $Z_{b}$ is the mean between-system connectivity of each association system to all sensory-motor systems. System segregation was then calculated based on the average mean within-system connectivity ($\overset{¯}{Z_{w}}$) and average mean between-system connectivity ($\overset{¯}{Z_{b}}$) values.

## Results

### The Locations of Prominent RSFC-Defined Boundaries are Consistent Across Age, Although Increasing Age is Associated With Less Parcellation Similarity Relative to Younger Adults

**Figure 1.**
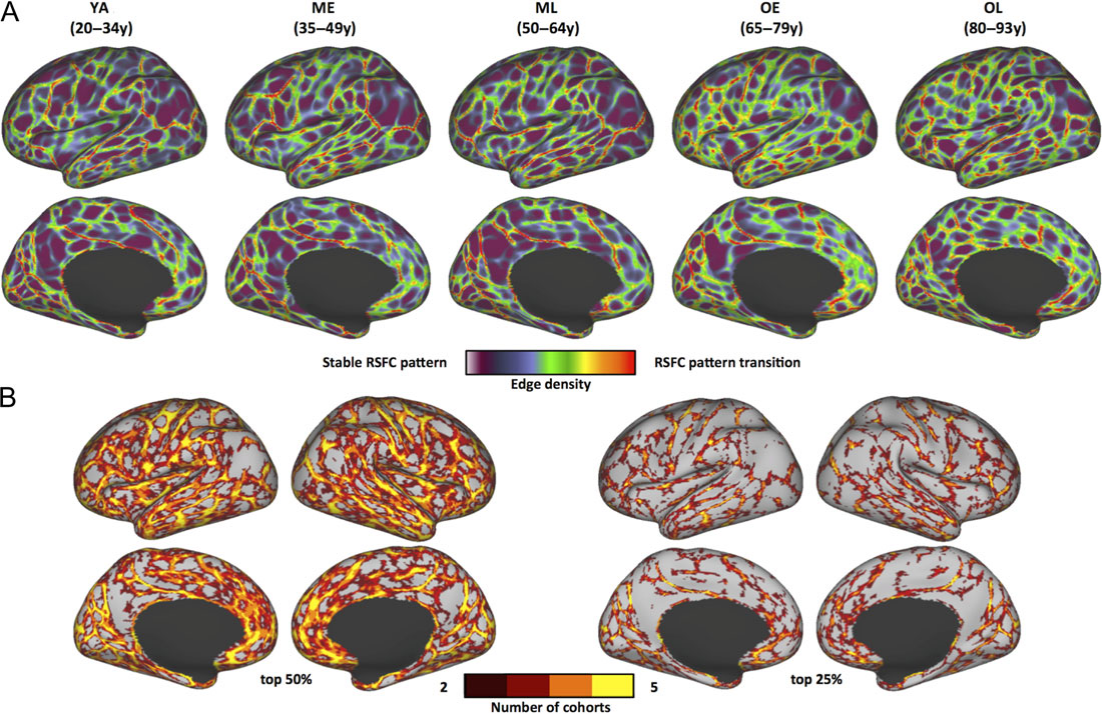
RSFC-based boundary maps exhibit consistent boundaries across adult cohorts in Dataset 1. (*A*) RSFC boundary maps of 5 age cohorts: brighter colors indicate higher probability of RSFC pattern transition (i.e., a putative area boundary). Darker colors correspond to vertices with stable RSFC patterns (i.e., less likely to be area boundaries). Lateral and medial views of the left hemisphere are depicted. (*B*) Conjunction images depicting locations where stronger boundaries are consistently identified across cohorts. Each individual cohort map from (*A*) was thresholded to its top 50% (left) and 25% (right) boundary map values, binarized, and summed to reveal boundary features that are consistently identified across at least 2 and up to all 5 cohorts.

RSFC-defined parcellation maps were derived from boundary maps that identify locations where RSFC patterns exhibit abrupt transitions across the cortical surface—these boundary maps delineate putative borders between brain areas (Cohen et al. 2008; Wig, Laumann and Petersen 2014; Gordon et al. 2016). Age cohort-specific boundary maps were computed for participants in Dataset 1 (Table 1) by applying this method to data averaged across groups of subjects that were sampled across the healthy adult lifespan. Visual inspection of boundary maps reveals that locations of cortical boundaries are similar across all cohorts in numerous locations, while other RSFC boundary features differ across one or more cohorts’ maps (Fig. 1*A*). Dice coefficients (spatial similarity indices) were used to quantify the similarity between the young adult (YA) boundary map relative to each non-YA cohort’s boundary map (ME, ML, OE, OL). All boundary maps were first thresholded to retain the strongest borders (top 1–100%, in steps of 1%; Fig. S2). The Dice coefficient between each non-YA cohort’s boundary map and the YA boundary map was significantly greater than null distributions built from randomly rotated versions of the YA map across thresholds (all *P*s < 0.01). However, there was a nominal decline of boundary similarity from younger to older cohorts, suggesting that functional boundaries tend to be less similar to younger adults with increasing age (e.g., for top 50% of the boundaries, $Z_{boundary\;maps\;[\mathrm{ME},\mathrm{ML},\mathrm{OE},\mathrm{OL}]}=\left[24.56,\;24.59,\;15.63,\;9.91\right]$, *P*s < 0.01). Despite the quantitative differences across cohorts, direct evaluation of border overlap revealed that many of the strongest borders were present in each of the 5 independent cohort maps, providing evidence that certain prominent area features do not differ across the healthy adult lifespan (Fig. 1*B*).

### Cohort-Specific Parcellations Exhibit Better Estimates of Parcel Quality Compared With the YA Parcellation Map

**Figure 2.**
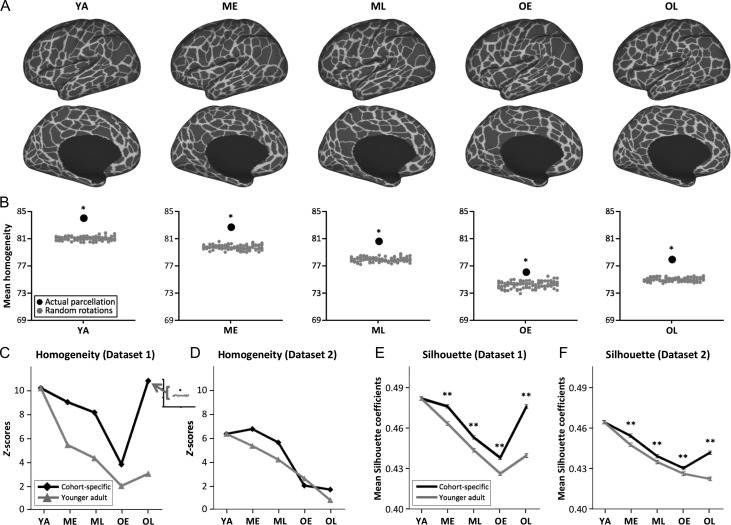
Cohort-specific functional parcellations of Dataset 1 derived from RSFC-based boundary detection are of high quality and provide a better “fit” to data from their own age range, compared with parcellation defined from younger adults. (*A*) Functional parcels of 5 age cohorts, calculated from their corresponding boundary maps. (*B*) For each cohort-specific parcellation, parcels exhibit significantly greater homogeneity of their vertices’ RSFC maps (black dots) relative to corresponding null model iterations (gray dots). (*C*) *Z*-scores of homogeneity values are plotted for cohort-specific parcellations and YA parcellation applied to each cohort’s dataset. The homogeneity of both cohort-specific and YA parcellations are significantly higher than their corresponding null models, however, cohort-specific parcellations provide a better representation of their corresponding data, as indicated by higher homogeneity *Z*-scores. The homogeneity values in Dataset 1 were evaluated from the same data as was used to derive the parcellations. (*D*) As in (*C*), except using a separate dataset (Dataset 2) for homogeneity evaluation. Cohort-specific parcellations exhibit higher homogeneity *Z*-scores than YA parcellation in ME, ML, and OL cohorts. (*E*, *F*) Mean Silhouette coefficients of cohort-specific parcellations are significantly higher than YA parcellation in both datasets, suggesting greater reliability of cohort-specific parcellations to define brain areas in their corresponding age ranges. Error bars indicate 95% confidence intervals of mean coefficients. Significance levels for *Z*-scores of homogeneity and 2-sample *t*-tests of Silhouette coefficient: *P < 0.01; **P < 0.001.

An area parcellation map was created for each age cohort from Dataset 1 by applying a watershed edge detection algorithm to the cohort’s RSFC boundary map (Fig. 2*A*). The total number of parcels identified in each parcellation map was comparable across cohorts, ranging from 468 to 522. And the numbers of parcels in left and right hemispheres were comparable across all cohorts (left hemisphere: 242 ± 13, right hemisphere: 239 ± 12). The difference in mean parcel surface area between cohorts was not significant (*F*_(4, 2395)_ = 2.32, *P* = 0.055; Table S1).

To determine whether the cohort-specific parcels represent valid estimates of functional parcellations (i.e., putative brain areas), it is important to examine whether parcels exhibit internal uniformity of the parcellation features (i.e., the parcels enclose sets of vertices with similar connectivity profiles) relative to null distributions. Parcel RSFC homogeneity was calculated using each parcel’s RSFC patterns across all constituent vertices (Laumann et al. 2015; Gordon et al. 2016). Mean homogeneity for each cohort’s parcellation was compared with an appropriate null model (RSFC signal homogeneity derived from randomly rotated parcellations of the same cohort; see Materials and Methods) to allow statistical evaluation. For each cohort’s parcellation map, mean homogeneity of the parcellation was significantly greater than its corresponding null model (all $Z_{homogeneity}s>3.84$, *P*s < 0.01; Fig. 2*B*).

A central hypothesis of the present report is that cohort-specific parcellations are better suited to their own data than a YA-defined parcellation. Accordingly, we examined whether YA parcellation applied to each non-YA cohort’s data would exhibit higher parcel homogeneity than cohort-specific parcellations. For each cohort, the YA parcellation exhibited homogeneity that was better than the null model (all $Z_{homogeneity}s>2.01$, all *P*s ≤ 0.02). Notably, however, the $Z_{homogeneity}$ derived from YA parcellation was consistently lower than that measured from cohort-specific parcellations in every case (Fig. 2*C*). This provides evidence that while parcellation defined from young adults may capture features of older cohorts’ data, it is less effective at capturing the precise functional divisions in older individuals.

One caveat to the observations noted above is that parcel quality evaluation (homogeneity testing) was performed on the same dataset used for parcel generation. As such, a second dataset was used to further evaluate cohort-specific parcel quality (Fig. 2*D*). Parcels defined from Dataset 1 were applied to RSFC patterns of Dataset 2 (the participant age distribution and composition of participants across cohorts in Dataset 2 was comparable to that of Dataset 1; Table 1). Results were consistent with those derived using Dataset 1: all cohort-specific parcellations were more homogeneous than their respective null models. Specifically, except for OL that showed a marginal effect (*P* = 0.06), each cohort exhibited a significant difference between their parcellations and their null models (all *P*s ≤ 0.03). We also observed a decline of $Z_{homogeneity}$ with increasing age, suggesting that the parcels in older cohorts are less homogenous than those of younger cohorts, possibly due to greater variability across participants within cohorts in Dataset 2. In comparison to YA parcellation, the cohort-specific parcellations exhibited greater $Z_{homogeneity}$ in ME, ML, and OL cohorts, supporting the observations for age-related differences in area parcellation. In addition, for the OL cohort, the homogeneity derived from applying YA parcellation to the OL data was not significantly better than the corresponding null model (*P* = 0.22). For the OE cohort, the $Z_{homogeneity}$ value for YA parcellation was comparable to that of the OE parcellation.

While homogeneity tests evaluate whether the signals within each parcel are uniform, measurement of the Silhouette coefficient quantifies parcellation quality by summarizing how similar the RSFC profile of each vertex is to other vertices in the same parcel relative to the vertices in all immediately adjacent parcels. In Dataset 1, the mean Silhouette coefficient of cohort-specific parcellations was significantly higher than the Silhouette coefficient of YA parcellation applied to each cohort’s data (*t*s > 10.05, *P*s < 0.001; Fig. 2*E*). This observation was replicated when the parcels were evaluated in Dataset 2 (*t*s >4.27, *P*s < 0.001; Fig. 2*F*). Collectively, the results suggest that cohort-specific parcellations exhibit better “fit” to data from their corresponding age range in comparison to YA parcellation, and provide evidence for some age-related differences in area parcellation.

### Evaluation of Spatial, Functional, and Anatomical Features of Boundaries and Parcellations Across Cohorts

**Figure 3.**
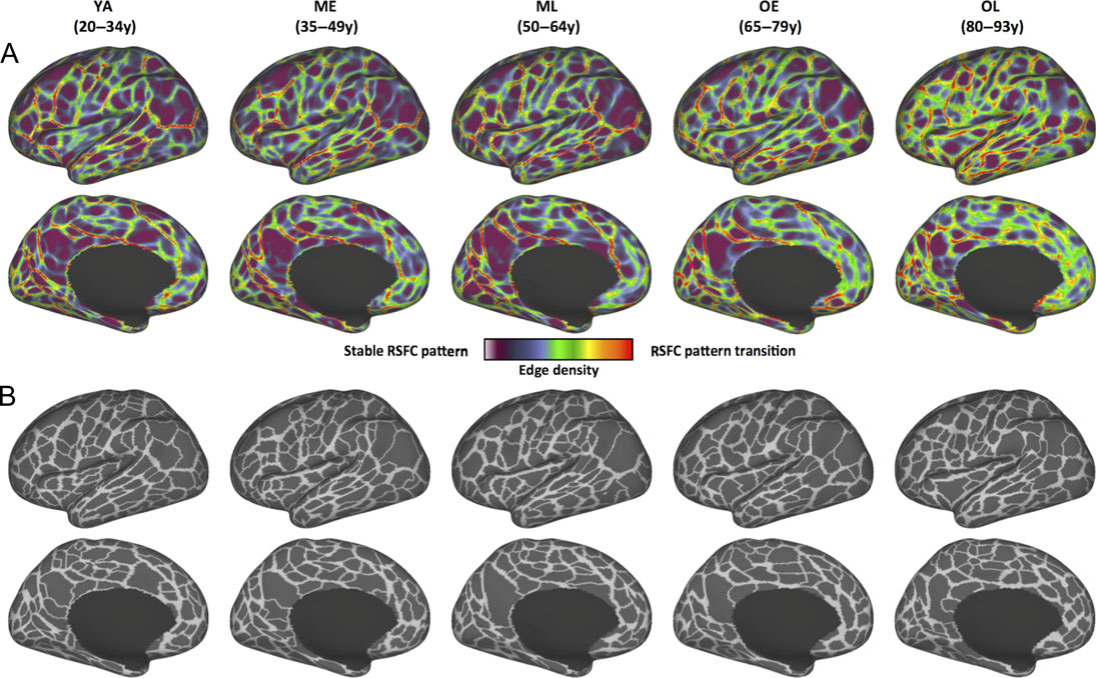
RSFC-based boundary maps and parcellation maps using the combined dataset. Datasets 1 and 2 were combined to create a final set of boundaries and parcellation maps. (*A*) RSFC boundary maps of 5 age cohorts: brighter colors indicate higher probability of RSFC pattern transition (i.e., a putative area boundary). Darker colors correspond to vertices with stable RSFC patterns (i.e., less likely to be area boundaries). (*B*) Functional parcels of 5 age cohorts, calculated from their corresponding boundary maps. Lateral and medial views of the left hemisphere are depicted here; see Supplemental Information (Fig. S4) for right hemisphere views and maps of boundary overlap.

While many RSFC-defined boundaries are consistently detected across age-cohorts, cohort-specific parcellations exhibit superior parcel quality (i.e., higher homogeneity and Silhouette coefficient) when applied to data from their own age range relative to applying the YA parcellation, implying possible age-related differences in the locations of some brain area boundaries. To maximize the amount of available data towards deriving more reliable parcellations, the 2 datasets were merged and parcellations from the combined dataset were created (Fig. 3 and Supplementary Results for additional details). In this combined dataset parcellation, the number of parcels was again comparable across cohorts, and the mean parcel surface area did not differ across cohorts (Table S2). This combined dataset parcellation was used for subsequent evaluation and comparison of anatomical and functional properties.

#### The Spatial Overlap of Parcels Predicts Similarity of RSFC Patterns Across Age

Visual inspection reveals that while some parcels are consistent across cohorts in terms of size, shape, and location, others look quite different (see Text S2, Fig. S4, and Table S2 for comparison of parcel number and size). The ARI was computed to quantify the spatial similarity between the YA parcellation map and each of the remaining cohort’s parcellation maps. In each comparison, the ARI was greater than its corresponding null model, indicating the spatial similarity between the YA parcellation and each non-YA parcellation was significant (all $Z_{ARI}s>9.05$, *P*s < 0.01). However, the ARI parcellation similarity statistic declined with increasing age ($Z_{ARI}$ for ME, ML, OE, and OL, respectively: 21.34, 16.72, 12.45, and 9.05), providing evidence that when compared with YA parcels, the spatial similarity of derived parcels exhibit decreasing similarity with increasing age.

**Figure 4.**
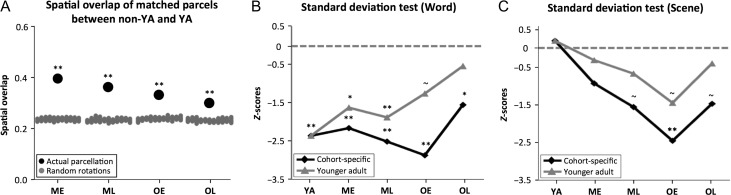
Spatial overlap of matched parcels between non-YA and YA cohorts declines with age; cohort-specific parcellations provide better estimates of task-evoked functional activity patterns relative to YA parcellation. (*A*) For each non-YA and YA cohort pair, the spatial overlap of matched parcels (black dots) are significantly higher than the spatial overlap of matched parcel rotations (the null model; gray dots). The distance between the black dot and gray dots, which was quantified by $Z_{overlap}$ (see Materials and Methods), declines across age cohorts suggesting decreasing spatial overlap of parcellations. (*B*) During a word judgment task, the variance (SD) of mean functional activity data within cohort-specific parcels is significantly lower than null models, whereas there is no significant difference between YA parcellation and its null model in older cohorts. The dotted line denotes *Z* = 0 where the SD of cohort parcels equals the SD of their rotations (i.e., parcellations would exhibit the same performance as their null models). (*C*) During a visual scene judgment task, statistically significant differences are only present for OE cohort-specific parcels, although *Z*-scores of cohort-specific parcellations are lower than YA parcellations applied to non-YA cohort task data. Significance levels for spatial overlap analysis and standard deviation test: **P ≤ 0.01; *P ≤ 0.05; ^~^*P* ≤ 0.1.

To more specifically map spatial consistency of parcels across cohorts, the spatial overlap between every parcel in each non-YA cohort’s parcellation map and every parcel in the YA parcellation map was computed, using maximal overlap percentage (see Materials and Methods). For every comparison (i.e., ME to YA, ML to YA, OE to YA, OL to YA), the mean overlapping percentage of matched parcels was found to be significantly higher than its corresponding null model (all $Z_{overlap}s>9.16$, *P*s < 0.01; Fig. 4*A*). However, the spatial distribution of this overlap varied across the cortical surface (Fig. S6C).

We hypothesized that parcels that are spatially similar to one-another would also exhibit similar patterns of RSFC, across cohorts. The RSFC profile of each parcel was calculated as the correlation between the mean time series of the parcel and the time series of all cortical vertices for each subject; the RSFC of the parcel was then averaged across all subjects of each cohort (see Materials and Methods). For all non-YA cohorts to YA-cohort comparisons, the RSFC similarity of spatially matched parcels was consistently high (0.691–0.869; see Materials and Methods for calculation) and significantly greater than corresponding null models (all *P*s < 0.01). However, $Z_{RSFC}$ declined with increasing age $\left(Z_{RSFC[\mathrm{ME},\mathrm{ML},\mathrm{OE},\mathrm{OL}]}=[11.52,\;8.18,\;7.90,\;5.28]\right)$, suggesting a divergence in RSFC patterns that paralleled their decreasing parcel overlap with increasing adult age. Consistent with this, the spatial overlap between parcels directly predicted RSFC similarity in each non-YA cohort to YA-cohort parcellation comparison (*r*s > 0.417; *P*s < 0.0001), indicating that parcels with greater spatial similarity also exhibit greater RSFC similarity across cohorts. Altogether, the results reveal that parcels matched based on spatial features are highly similar in their functional fingerprints as well. The analysis of large-scale RSFC network patterns follows in a later section.

#### Cohort-Specific Parcellations Provide Better Convergence With Task-Related Functional Activity

Previous studies have demonstrated a convergence between RSFC boundaries and areas defined by task-evoked activity (Wig, Laumann and Petersen 2014; Laumann et al. 2015; Glasser et al. 2016; Arslan et al. 2018). We hypothesized that relative to the parcellation defined from younger adults, cohort-specific RSFC parcellations would provide better estimates of task-related variance for data from their corresponding age cohorts. Specifically, we determined whether cohort-specific parcels would exhibit greater homogeneity in their cohort’s mean functional (BOLD) activity; this was tested by computing and comparing the SD values of task activity within parcels. For the word judgment task, the SD of beta values in cohort-specific parcellations were significantly lower than their corresponding null models (all *P*s ≤ 0.05; Fig. 4*B*), and relative to the YA parcellation (which did not significantly differ from their null models for older cohorts, i.e., OE and OL). This suggests that relative to the YA parcellation, cohort-specific parcellations are better able to capture uniform task-evoked signals within parcels. For the scene classification task, the difference between cohort-specific parcellations and their null models was consistently lower than that of YA parcellation, although these differences were only significant in the OE cohort (Fig. 4*C*). Curiously, the SD of beta values for the YA parcellation applied to younger adult data was not lower than the corresponding null models, suggesting that younger adults exhibited a high degree of variance in their task-activation maps during scene classification.

#### Differences in Brain Structure and Anatomical Alignment Explain a Significant Portion of the Parcellation Differences Across Age

**Figure 5.**
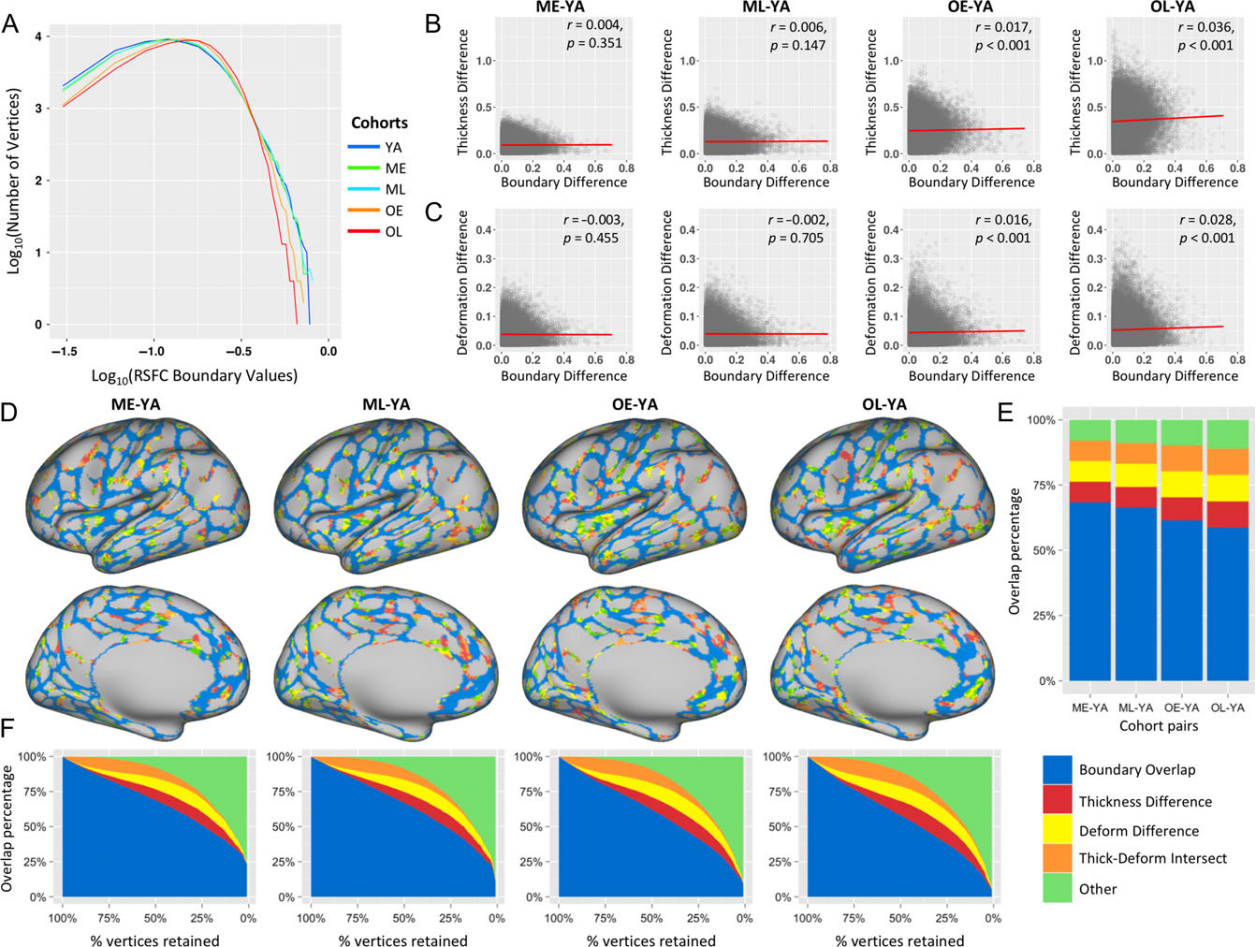
Locations that exhibit boundary differences (non-YA cohorts vs. YA) largely coincide with locations that exhibit differences in cortical thickness and anatomical alignment across cohorts. (*A*) depicts the boundary value distribution of 5 cohorts; both *x* axis (boundary values) and *y* axis (number of vertices) were transformed using a logarithmic function for better visualization. Relative to the younger adult cohort, older adult cohorts (OE and OL) exhibit more vertices corresponding to medium probability RSFC boundary values, and less vertices exhibiting both higher (more likely to be area borders) and lower probability boundaries (more likely to be areas). (*B*) Boundary differences are significantly correlated with thickness differences in older cohorts (OE and OL), but not middle-age cohorts (ME and ML). (*C*) Boundary differences are significantly correlated with deformation differences for OE and OL, indicating that the vertices where there are greater boundary differences are also the locations of greater deformation differences for older cohorts (see Text S3 for details). (*D*) The similarity of the boundaries between each non-YA and YA cohorts were calculated based on spatial overlap across multiple thresholds of maps. At a threshold of 50%, the majority of the boundaries overlap with each other (vertices in blue), indicating high similarity between the 2 cohorts across the cortex. For the locations where boundaries were present in the YA cohort but absent in the non-YA cohorts, the majority of those vertices either exhibit between-cohort differences in cortical thickness (red), anatomical alignment (i.e., deformation; yellow), or both (orange). Vertices colored green correspond to locations where a boundary is present in the YA map but absent the non-YA map, and did not exhibit between-cohort differences in neither cortical thickness nor anatomical alignment. The distribution and proportion of these relationships is plotted in (*E*). While the majority of YA vertices exhibit overlap with non-YA cohort boundaries, the remainders are largely locations that exhibit differences in the deformation map and cortical thickness maps (all thresholded to retain the top 50% of vertices). (*F*) The correspondence between boundary overlaps and thickness/alignment differences exhibits similar patterns across thresholds (top 1–100% of possible vertex values, in steps of 1%).

It’s visually clear that while many of the parcellation boundaries are consistent across cohorts (Fig. 1*B* and Fig. S4C), there is also increasing variability with increasing age. Consistent with these impressions, the spatial correlation between each cohort’s unthresholded boundary map was significant when compared with the YA boundary map (*r*_[ME–YA,ML–YA,OE–YA,OL–YA]_ = [0.529, 0.448, 0.334, 0.248], *P*s < 0.0001), although the percent variance explained decreased with increasing age. Specifically, increasing age was accompanied by a greater number of locations (vertices) corresponding to medium probability RSFC transition zones (less likely to be boundaries), and a fewer number of locations (vertices) both corresponding to higher probability transition zones (more likely to be boundaries) and to lower probability RSFC transition zones (more likely to be areas; Fig. 5*A*). We hypothesized that some of the differences in parcellation across age may be due to underlying age-related differences in brain anatomy (Good et al. 2001; Apostolova et al. 2012). Relatedly, the increasing variability in cortical structure across adult cohorts (Magnotta et al. 1999; Sowell et al. 2003; Raz et al. 2005) could also result in differing variability of anatomical alignment to the surface atlas space, which would impact estimates of group-derived parcellation features. To directly evaluate these hypotheses, vertex-wise boundary map differences were quantified across cohorts and compared with vertex-wise differences of both mean gray-matter cortical thickness and mean anatomical deformation.

To determine whether the differences in boundary maps could be explained by the variability of cortical thickness and/or anatomical deformation, the vertex-wise difference between each cohort’s boundary map and the YA boundary map was computed. For each cohort, the absolute value of the vertex-wise boundary map difference (non-YA cohort – YA cohort) was entered into a multiple regression analysis as a dependent measure, and the absolute value of the vertex-wise cortical thickness difference and vertex-wise anatomical deformation difference were entered as independent predictors. The models were significant for both of the older-age cohorts (OE: *F*_(2,59409)_ = 16.22, *P* < 0.0001; OL: *F*_(2,59409)_ = 59.46, *P* < 0.0001), but not the middle-age cohorts (ME: *F*_(2,59409)_ = 0.72, *P* = 0.487; ML: *F*_(2,59409)_ = 1.13, *P* = 0.322). For the older cohorts, both the main effect of cortical thickness difference (OE: *t*_(59409)_ = 4.23, *P* < 0.0001; OL: *t*_(59409)_ = 8.60, *P* < 0.0001; Fig. 5*B*) and deformation difference (OE: *t*_(59409)_ = 3.78, *P* < 0.0001; OL: *t*_(59409)_ = 6.32, *P* < 0.0001; Fig. 5*C*) were statistically significant. The interactions between cortical thickness differences and deformation differences on boundary map differences were not significant. The results suggest that for older cohorts, age-related differences in cortical thickness and anatomical alignment variability explain some of the boundary differences between older and young adults at a vertex level.

An alternative approach for performing the above evaluations is to first threshold the boundary maps (Fig. 1*B*), determine the amount of overlap between each non-YA cohort with YA, and then examine if the nonoverlap coincides with locations where the cohort and YA differ in cortical thickness, anatomical deformation, or their intersection. To visually illustrate this, we present each cohort’s thresholded boundary map (at 50%), and color each cohort’s boundaries based on whether they overlap with YA boundaries (blue color in Fig. 5*D*). In many cortical locations where there is an absence of overlap between a given cohort’s boundaries and YA boundaries, we observed differences in cortical thickness, anatomical deformation, or their intersection (Fig. 5*E*). This was consistent across a broad range of thresholds (Fig. 5*F*; see Fig. S7 and Text S3 for additional results).

Overall, these results supported the observation that the majority of area boundaries are consistent when comparing the younger adult boundary map to each of the non-YA cohort’s boundary map, and that a large portion of the nonoverlap can be accounted for by differences in cortical structure and within-cohort variability in anatomical alignment.

### Large-Scale Network Analysis of the Adult-Lifespan Using Cohort-Specific Nodes

#### Resting-State Community Organization is Similar Across Age

**Figure 6.**
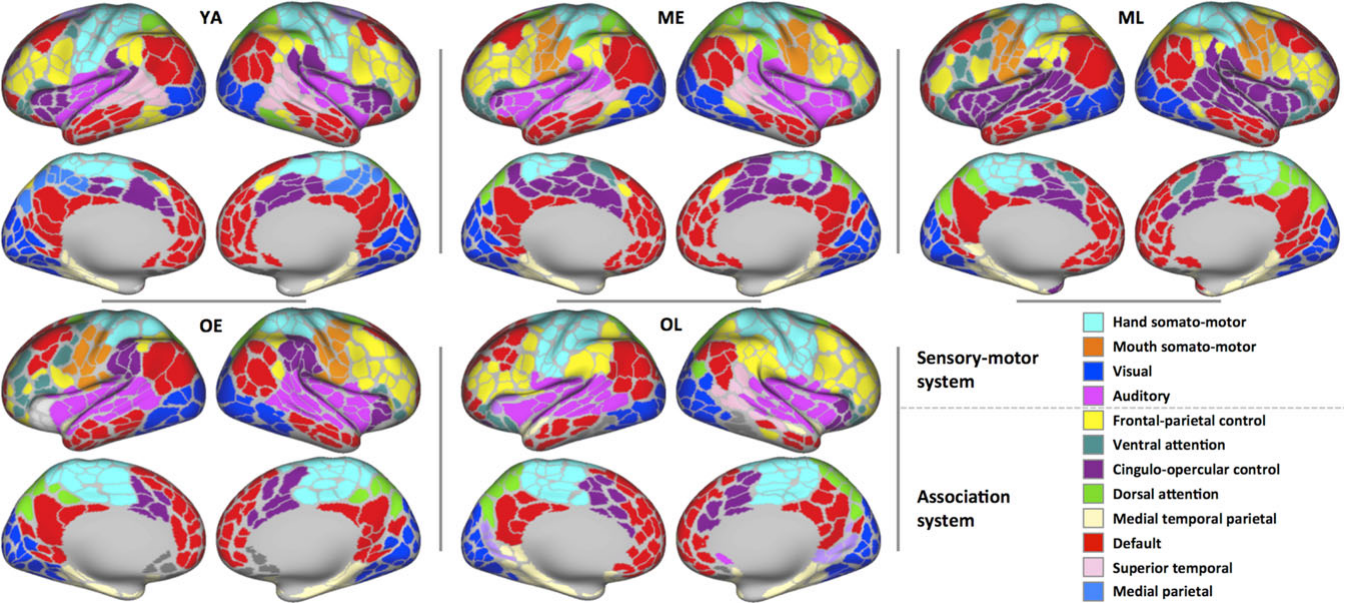
Community detection using cohort-specific parcellations reveals the presence and consistency of large-scale systems across the healthy adult lifespan. Cohort-specific parcels were used as nodes to model RSFC networks within each cohort across a range of edge-density thresholds (see Supplementary text for details), revealing a consistent topography of large-scale systems. Commonly identified systems are labeled as in Power et al. (2011).

We have previously reported that network community structure is comparable across the adult lifespan (Chan et al. 2014, 2017). These observations were based on graphs built using nodes defined from young adults. Although older cohorts’ parcellations are similar to YA parcellation, using cohort-specific parcels as brain network nodes may reveal previously unidentified differences in functional network architecture across the adult lifespan. Using a consensus community-detection approach similar to previous literature (Gordon et al. 2016; Chan et al. 2017), subnetworks (communities) for each cohort were calculated across a range of edge-density thresholds using each cohort-specific parcellation as their brain network nodes (Fig. 6; see Fig. S8 for additional description of community assignments). Commonly described functional systems (Power et al. 2011; Yeo et al. 2011) were identified for each cohort across the adult lifespan. Furthermore, the community detection revealed highly similar network structures across cohorts; for each of the 10 major systems depicted in Figure 6, the spatial similarity between the YA community map and each non-YA community map was significantly greater than the corresponding null models, suggesting significant spatial overlap in large-scale brain systems across the adult lifespan (all $Z_{system}s>3.09$, *P*s ≤ 0.04). For these analyses, the mouth and hand somatomotor systems were combined (the mouth somatomotor system being separated from the hand somatomotor system in ME, ML and OE cohorts when the graphs were sparser, but this division was absent in YA and OL); the auditory community was not detected in the ML cohort, thus it was not included in this comparison.

#### System Segregation Decreases With Increasing Age Using Cohort-Specific Nodes

**Figure 7.**
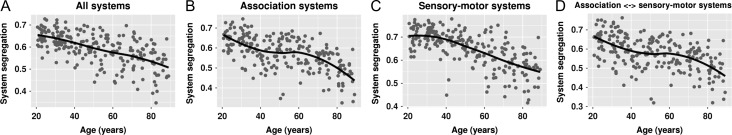
Using cohort-specific nodes, system segregation is negatively correlated with age. (*A*) In line with our previous findings (Chan et al. 2014), here using systems defined by cohort-specific community detection applied to graphs built using cohort-specific parcels as nodes, the system segregation exhibits an age-related decrease along the healthy adult lifespan. The segregation of (*B*) association systems, (*C*) sensory-motor systems, and (*D*) between association and sensory systems exhibited cubic relationships with age. Each point represents an individual; the line represents a locally weighted scatterplot smoothing (LOESS) curve of each relation (see Results for detailed statistics).

Cohort-specific parcellations that are able to define areas with greater precision should provide more accurate estimates of large-scale network properties. We applied cohort-specific parcellations to each cohort’s RSFC data to evaluate whether more accurate node definition alters the observed relationships between age and a measure of subnetwork organization: system segregation (Wig et al. 2011; Chan et al. 2014). System segregation characterizes the separation of the brain’s subnetworks by comparing connectivity within and between systems. Increasing age is associated with decreasing segregation of brain systems, although the observations thus far have been based on using nodes defined in young adults (Betzel et al. 2014; Geerligs et al. 2015; Chan et al. 2014; for review see Wig 2017). As accurate network modeling necessitates biologically plausible and accurate nodes (Smith et al. 2011; Wig et al. 2011), it is possible that the reports of age-accompanied desegregation have been due to decreased accuracy of sampling the underlying node features with increasing age; using cohort-specific nodes could address this possibility. Networks using the cohort-specific node set confirmed the previously reported decrease of network segregation across age (*r* = −0.594, *P* < 0.001; Fig. 7*A*). Examining distinct types of systems (i.e., sensory-motor and association systems) revealed that the segregation of both types of systems exhibited a cubic relationship with age (sensory-motor systems: *F*_(3,218)_ = 63.8, *P* < 0.001; association systems: *F*_(3,218)_ = 46.99, *P* < 0.001; Fig. 7*B*,*C*), as did the segregation of sensory-motor systems from association systems (*F*_(3,218)_ = 30.38, *P* < 0.001; Fig. 7*D*). These cubic age-related relationship patterns were not observed in our initial report. All together, these observations demonstrate that age-associated reductions in system segregation are not a consequence of age-biased sampling and labeling of brain network nodes or their associated systems, respectively.

## Discussion

RSFC-based boundary mapping methods were used to derive area parcellations for cohorts of individuals across the adult lifespan. Close examination of the boundaries across cohorts revealed that the strongest parcellation boundaries were largely consistent across the adult lifespan, suggesting a large degree of age-invariance in the spatial location of prominent area features. Despite the large degree of overlap, the spatial similarity of cohort-specific boundaries relative to YA boundaries decreased with increasing age. These differences in spatial similarity were closely coupled with differences in brain structure and anatomical alignment between the older adult cohorts and the younger adult cohort, providing evidence for plausible sources of the differences in RSFC-defined parcellation-features across age. Parcels defined from the boundary maps of each cohort exhibited greater homogeneity of their RSFC patterns relative to corresponding null models, providing support for delineation of functionally distinct parcels that may correspond to dissociable brain areas. While the parcels were similar to each other in terms of parcel number, size, and position, cohort-specific parcellations exhibited more homogeneous and representative estimates of resting-state and task signals from their own age range, relative to the parcels defined from younger adults. Using the cohort-specific parcellations to generate cohort-specific nodes used in graph-based large-scale RSFC network analysis demonstrated the presence and consistency of multiple network communities that align with major functional systems, in each age cohort. In addition, using cohort-specific nodes confirmed previous observations (Chan et al. 2014) that brain systems exhibit decreasing segregation with increasing age across individuals. Altogether, the present results provide an initial demonstration for age-accompanied similarities but also variation in large-scale cortical organization at the level of areal features, and their resultant network structure. The collective observations have both theoretical and practical implications towards numerous issues relevant for the neuroscience of aging and cortical organization.

### Many of the Differences in Area Parcellation Across the Adult Lifespan are Related to Differences in Brain Structure and Anatomical Alignment

Before discussing further the observed differences in parcellation with increasing age, it is important to acknowledge the similarities in parcellation that are prevalent across the adult lifespan. Support for this idea is established across numerous observations: 1) comparing younger adult parcels to each of the cohort-specific parcels reveals greater spatial similarity than expected by chance (Fig. 4*A*); 2) the locations of many of the strongest borders are consistent across age cohorts (Figs 1*B* and 5, and Fig. S4C); and 3) the spatial topography of large-scale RSFC systems defined using cohort-specific parcels exhibit high similarity across age (Fig 6). Collectively, these results suggest that the overall spatial organization of area parcellation is largely maintained across the healthy adult lifespan. This conclusion provides support for making direct spatial comparisons of anatomical and functional features across cohorts of adults at different ages.

Despite the similarities in parcellation noted above, our results provide evidence that there also exist distinctions in RSFC-defined area parcellation across different age cohorts that are important to consider. Direct matching of boundaries and parcel shapes revealed progressively greater divergence from the younger adult map with increasing age; for example, older adult cohorts exhibited a greater number of medium-strength RSFC-defined boundaries and a fewer number of stronger RSFC-defined boundaries, relative to the younger cohorts. Although we employed strict preprocessing procedures to minimize the potential influence of head motion on RSFC signals (Satterthwaite et al. 2013; Power et al. 2014), it is possible that differences in residual head motion across cohorts contributed to the observed parcellation differences (particularly in older age cohorts (Van Dijk et al. 2012; Savalia et al. 2017)). While supplemental analysis revealed that residual head movement may impact parcel quality, this aspect of the data quality could not fully explain the between cohort differences (Text S1 and Fig. S3). We hypothesized that some of the age-related variation in parcellation was associated with changes in brain anatomy that accompany healthy aging; this hypothesis was supported by identifying the contribution of differences in cortical gray matter thickness and anatomical alignment to boundary variability when comparing older cohorts to younger adults.

Numerous techniques have been developed to minimize the impact of individual differences on brain alignment (Fischl et al. 1999; Buckner et al. 2004; Van Essen et al. 2012) and we employed considerable steps to ensure precise estimates of gray matter tissue segmentation (Savalia et al. 2017) which were used in subsequent surface-based alignment (Dale et al. 1999; Fischl et al. 1999; Ségonne et al. 2005; Van Essen et al. 2012). This likely contributed to the detection of consistent boundaries across cohorts and homogenous parcels relative to null models. However, gray matter cortical thickness along with other aspects of anatomical morphometry also exhibits increasing variability with increasing age (Sowell et al. 2003); these differences compromise anatomical alignment for older age groups (Fischl and Dale 2000). In keeping with this, our own supplemental analyses revealed that increasing age was associated with increasing variability in anatomical deformation (see Text S3 for results). At the regional level, locations that exhibited thickness differences when comparing younger to older adults also exhibited differences in anatomical deformation (although these 2 measures were not perfectly correlated suggesting some uniqueness, see Supplemental Information for results). Critically, these locations also coincided with locations where there was an absence of detected boundaries when comparing younger adult parcellation to older adult parcellations (Fig. 5) and also with locations where boundaries were present in older but not younger adults (Fig. S7), thus, providing evidence that some of the parcellation differences may stem from differences in brain anatomy.

Cortical thinning exhibits different trajectories for distinct brain areas along the healthy lifespan (Sowell et al. 2003; Salat et al. 2004); for example, locations typically categorized as being part of “association cortex” (Mesulam 1990) typically exhibit the greatest age-related anatomical change (Resnick et al. 2003; Raz et al. 2005; Fjell et al. 2009). At a cellular level, differences in cortical thickness can be attributed to shrinkage and loss of neurons (Kril et al. 2004), or reduction in dendritic arborization (Morrison and Hof 1997). Age-related cortical thinning has also been found to relate to differences in genetic organization patterns (Fjell et al. 2015), which may undermine the information processing and storage in neurons (Small et al. 2002, 2004). These observations have led to ideas regarding age-related differences in the function of areas (Cabeza et al. 1997; Grady et al. 1998; Reuter-Lorenz et al. 2000). Patterns of RSFC are thought to represent a history of coactivation patterns (Wig et al. 2011) and functional activation patterns differ with increasing age (Reuter-Lorenz et al. 2000; Park et al. 2004; Chan et al. 2017). These functional differences may result in either reduced ability to detect strong cortical boundaries between adjacent regions (under-parcellation) or detection of transitions within areas that are exhibiting increased differentiation of RSFC patterns in the older cohorts (over-parcellation). Further, as RSFC patterns are constrained by white matter connections (Honey et al. 2009), the variation in white matter volume (Bartzokis et al. 2001; Allen et al. 2005) and integrity (for review see Kennedy and Raz 2015) that accompanies healthy aging might also contribute to the observed distinctions in RSFC-defined parcellation maps observed here, even in locations where there were no observed differences in cortical thinning or alignment (i.e., the “green” vertices in Fig. 5). Related to all this, a number of important earlier studies of regional activation patterns in aging demonstrated differences in the spatial extent of task-related activation (Grady et al. 1998; Reuter-Lorenz et al. 2000). While these observations were unable to adjudicate the possible source of the differences, our results suggest that the activation differences may relate to differences in spatial alignment of areas; an alternate possibility is that the activation differences are due to differences in the strength of area function/borders across age, possibly in relation to experience-related changes in cortical function. Naturally, these different explanations are not mutually exclusive and may be related. It remains to be seen whether and how any of the inconsistent boundaries in the present report correspond to previously described functional distinctions.

### The Spatial Topography of Large-Scale Networks is Consistent Across the Healthy Adult Lifespan

Age-associated similarities were not limited to areal parcellation; parcel-wise maps of network communities were spatially similar across age cohorts as well. In each age cohort’s organized networks, we identified major RSFC functional systems that have been previously reported (e.g., the default system, cingulo-opercular control system, visual system) (Power et al. 2011; Yeo et al. 2011). Examination and comparison of large-scale network organization across aging and disease has typically relied on using YA parcellation to construct network representations (Chan et al. 2014; Buckley et al. 2017). A tacit assumption of these approaches is that the nodes are equally appropriate across all participants. However, without direct evidence supporting this assumption, any individual differences observed in network organization or its properties may be influenced—at least in part—by systematic bias in sampling biologically appropriate and accurate nodes across individuals (Wig et al. 2011). As we have attempted to minimize this source of error, our results provide evidence that large-scale system organization is comparable across adult age.

We have previously provided evidence for the presence of large-scale systems across age (Chan et al. 2014, 2017), although those reports did not examine or directly compare the spatial topography of those systems. The present findings demonstrate that large-scale brain systems are maintained across the adult lifespan, which stands in contrast to previous studies that have reported qualitative differences in the organization of the brain’s subnetworks when comparing younger to older adults (Meunier et al. 2009; Geerligs et al. 2015). While direct examination of all the factors that differ across the studies is beyond the scope of the present report, we suspect that a large source of these differences can be attributed to differences in preprocessing techniques (e.g., inadequately removing motion-contaminated frames using appropriate thresholds for frame removal) and distinctions in methods of node-definition.

A number of smaller qualitative differences in community assignment were observed across age. For the most part, this was due to differences in community assignment observed across different edge density thresholds. For instance, nodes in left prefrontal cortex were assigned to the frontal–parietal community across most thresholds in the OL cohort, but broke off to form an independent community across sparser graph-density thresholds (see Fig. S8). This observation speaks to the distribution of connection strengths that form the sub-graph, and also converges with previous findings that describe a weakening of within system connections in older age (Andrews-Hanna et al. 2007; Damoiseaux et al. 2008; Chan et al. 2014). Despite these nuanced differences that need to be explored further, our approach and recommendation involves examining community assignments across a range of thresholds to ensure that an inferred pattern is not constrained to a specific edge-density threshold or parameter.

### Cohort-Specific Nodes Reveal Relationships Between Aging and System Segregation

Cohort-specific parcels were used as network nodes, and together with their cohort-specific community assignments, measures of system segregation were calculated for each participant. The size and number of parcels (nodes) was highly similar across cohorts (Table S2); the small differences in node number are in a regime where graph measures have been shown to remain relatively stable when functionally defined parcels are examined (Arslan et al. 2018).

Despite the similarity of communities identified across cohorts, age-related differences in network organization were observed in the current report, as revealed by decreased system segregation along the adult lifespan. This is in accordance with previous findings using young adult nodes, demonstrating age-related differences in brain-wide segregation/modularity (for review, see Wig 2017). Specifically, we have previously argued that decreased segregation reflects a type of “de-differentiation” of the brain’s functional systems within the network (Chan et al. 2014), that these differences in network organization relate to the brain’s functional activity (Chan et al. 2017) and are moderated by an individual’s environment (Chan et al. 2018). Here we provide evidence that the age-segregation relationship is not likely due to biased node sampling in that it is still present using cohort-specific nodes.

### Cohort-Specific Parcellations Provide Better Estimates of Functionally Distinct Areas Than Parcellations Defined From Younger Adults

Well-defined functional areas permit accurate characterization of brain features. Parcels can serve as a priori region of interests (ROIs) that contain uniform brain signals, and in doing so minimize the between-subject variance of task and connectivity estimates within a cohort. We and others have demonstrated that parcellations defined by resting-state data can define areas that are in good agreement with task-evoked activity patterns in young adults (Wig, Laumann and Petersen 2014; Laumann et al. 2015; Glasser et al. 2016; Gordon et al. 2016; Arslan et al. 2018). The present observations support these observations. They further demonstrate that while younger adult parcellation can be applied to older adult cohorts, cohort-specific parcellation is feasible and may be necessary for accurate characterization of individuals who exhibit differences in their brain anatomy in order to ensure accurate areal localization. To review, our processing stream utilized surface-based registration procedures that minimize the bias of anatomical variability and cross-participant brain alignment. Notwithstanding this important processing step, additionally employing cohort-specific parcellations provided enhanced area localization relative to the application of YA parcellation to non-YA subjects, as evidenced by the greater parcel homogeneity of RSFC patterns and lower parcel variance of task-signal estimates. Collectively, these results highlight the advantages of defining functional areas at an age-appropriate level.

While we have focused on the differences in area parcellation across the adult lifespan, the approach has implications that extend beyond these specific comparisons and domains of analysis. Both anatomical and functional variability are prevalent across individuals (Devlin and Poldrack 2007; Van Essen and Dierker 2007; Amunts and Zilles 2015; Laumann et al. 2015). Numerous registration procedures have been developed to overcome the anatomical source of intersubject variance (Fischl et al. 1999; Buckner et al. 2004; Van Essen et al. 2012). An appreciation that the brain’s functional processing units need not respect anatomical landmarks has led to the additional development of procedures that align maps using “hyperalignment” of functional signals (Haxby et al. 2011), using multimodal data (Robinson et al. 2014) and subject-specific ROIs (Saxe et al. 2006). Here, we demonstrate that a parcellation based on younger adults may inaccurately prescribe the functional boundaries of older adults, which can both impact estimates of brain function and anatomy and may also lead to inaccuracies in downstream applications of these tools (e.g., defining nodes in a large-scale brain network; Wig et al. 2011). More broadly, this collective work continues to challenge the assumption that areal organization is identical across individuals (Caspers et al. 2006). While individualized parcellation may not be feasible due to data limitations (Laumann et al. 2015; Gordon et al. 2017) associated with collecting large samples of special populations, the present results encourage continued development and examination of potential area parcellation differences across different segments of the entire lifespan (i.e., including children and adolescents), and health status (e.g., patients vs. controls).

### Data Limitations and Future Directions

Cohort-specific parcel homogeneity decreased with increasing age, suggesting that while cohort-specific parcellations are better than the YA parcellation for older adults, their “fit” to their own data is poorer. This observation could not entirely be explained by residual motion (Text S1 and Fig. S3), and may be a consequence of greater between-subject variability (e.g., anatomical and functional features within cohort samples) of increasing age, as revealed by the contribution of brain misalignment to boundary variability. Indeed, relative to younger adults, older subjects exhibit greater variability in measures of anatomy and functional activity (Raz et al. 2005; Reuter-Lorenz and Cappell 2008).

Our cross-validation employed incorporation of a second dataset that was comprised of a subset of participants that were also present in the first dataset. While having access to the second dataset allowed us to take important steps towards evaluating the replicability of parcellations, the second dataset was not entirely independent in terms of subject composition within each cohort. Additional work will be important to understand the generalizability of our cohort-specific parcellations.

Related to the above, while the parcellations maps were largely derived from cross-sectional data, additional considerations present possible longitudinal applications of adult aging parcellations. Direct comparison of parcellations reveals that adjacent age-defined cohorts are most similar to one another (Fig. S6B). The potential parcellation mismatches are relatively minimal within the time-frames that are likely to be assessed with longitudinal data analysis, compared with potential parcellation errors that might be introduced when applying a young adult parcellation to an older adult parcellation, for example. Based on this, one strategy would be to apply the participant’s original parcellation (based on their age at time of first data acquisition) to all subsequent longitudinal estimates. However, as longitudinal analyses begin to reveal potential differences in aging networks, additional consideration and evaluation of this important application of parcellation will be required.

A number of processing decisions imposed constraints on the types of analyses we conducted and the breadth of the conclusions that can be established from the present dataset. Since the central aim of our report was to examine parcellation and organization of the brain across a wide age range, our principle focus in data processing was to minimize sources of age-related variation in factors known to influence resting-state (Satterthwaite et al. 2013; Power et al. 2014) and anatomical (Alexander-Bloch et al. 2016; Savalia et al. 2017) signals. This decision inevitably resulted in data loss following our conservative QC and data cleaning procedures (both within a subject, but also of whole subjects who did not meet our pre-established criteria following data processing). However, the inherent trade-off that accompanies the choice of analyzing a greater amount of contaminated data versus a lesser amount of clean data was balanced by leveraging the availability of a large number of participants that were densely and relatively uniformly sampled across decades of adult life. Although our processing choices have allowed us to create cohort-defined parcellations in multiple datasets, it will be important to evaluate the observations throughout the report additionally using individual parcellations (Wig, Laumann, Cohen, et al. 2014; Laumann et al. 2015; Gordon et al. 2017), when the appropriate data become available. This approach may also minimize the issue of misalignment due to the nature of individual studies.

It is also important to recognize that functional areas defined by RSFC boundary mapping do not always overlap with architectonic divisions (Wig, Laumann and Petersen 2014). For example, RSFC transition zones are present within primary somatosensory, motor, and visual areas; these borders exhibit close correspondence with topographic distinctions (e.g., representations of mouth vs. hand, central vs. peripheral visual fields) (Wig, Laumann and Petersen 2014; Gordon et al. 2016). Conversely, there exist area borders where RSFC-defined transitions may not always be detected (e.g., the division between somatosensory and motor areas along the precentral/postcentral gyrus). All together these observations impose constraints on our interpretation of the parcellation differences observed here and their correspondence to potential area differences. Incorporating additional features of area definition and identifying the convergence of parcellations across modalities will be important for confirming the existence and nature of area parcellation differences across age (Glasser et al. 2016; for discussion see Wig et al. 2011; Eickhoff et al. 2018).

## Supplementary Material

Supplementary Data
