# Supplementary material for: Functional Parcellation of the Cerebral Cortex Across the Human Adult Lifespan
Source: Cereb Cortex. 2018 Oct 11;28(12):4403–23. doi: 10.1093/cercor/bhy218 (PMC6215466; doi:10.1093/cercor/bhy218)
Supplement: Supplementary Data [file bhy218_agingparcellation_si.docx]

**Functional Parcellation of the Cerebral Cortex Across the Human Adult Lifespan**

**Supplemental Information**

Liang Han^1^, Neil K. Savalia^1,2^, Micaela Y. Chan^1^, Phillip F. Agres^1^, Anupama S. Nair^1^ & Gagan S. Wig^1,3^

^1^Center for Vital Longevity and School of Behavioral and Brain Sciences, University of Texas at Dallas, Dallas, TX, 75235, USA

^2^ Yale University School of Medicine, New Haven, CT, 06520, USA

^3^Department of Psychiatry, University of Texas Southwestern Medical Center, Dallas, TX, 75390, USA

**Supplemental Information**

**Figure S1** Method for generating cohort-based parcellation maps using bootstrap-sampling of participants.

**Figure S2** Dice coefficients of boundary maps between YA with each non-YA cohort using Dataset 1.

**Table S1** Number of parcels and mean parcel area of the parcellations for each of the 5 cohorts using Dataset 1.

**Text S1** Relationship between head motion residuals and cohort-based parcellation quality.

**Figure S3**  Residual head motion and differences in data amount relate to part of the differences in parcellation quality across cohorts.

**Text S2** Description of parcellations derived from the combined dataset.

**Figure S4** The boundary maps, parcellation maps and boundary conjunction maps using the combined dataset.

**Table S2** Number of parcels and mean parcel area of the parcellations for each of the 5 cohorts using the combined dataset.

**Figure S5** The homogeneity and Silhouette coefficients of the parcellations using the combined dataset.

**Figure S6** The spatial similarity of boundary and parcellation maps using the combined dataset.

**Text S3** Relationship between variability of anatomical alignment, cortical thickness differences and boundary differences.

**Figure S7** Spatial consistency between each cohort’s boundary variability and brain anatomical differences.

**Figure S8** The un-altered community assignments for the 5 cohorts.


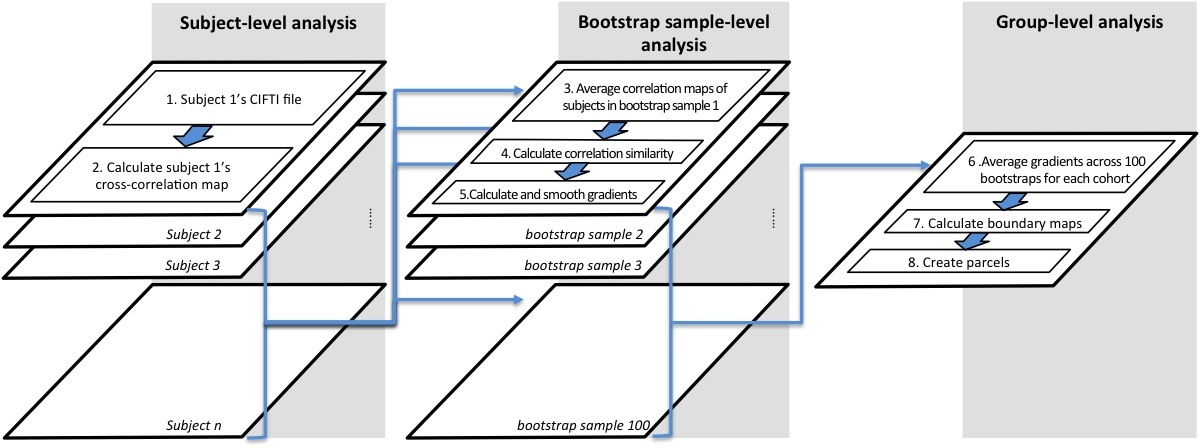


**Figure S1.** **Method for generating cohort-based parcellation maps using bootstrap-sampling of participants.** 1) Using each participant’s CIFTI file, 2) the cross-correlation maps for all surface vertices were calculated (32k × 64k; i.e. the correlation of the vertices in one hemisphere to the vertices in both hemispheres). 100 bootstrap samples (resampled with replacement) were created for each age cohort; in each sample the number of participants was equivalent to the original cohort sample size. 3) The mean correlation maps were created by averaging across the participants in each bootstrap sample, producing a 32k × 64k matrix for each bootstrap sample. 4) The vertex-wise RSFC similarity matrix was derived by calculating the pairwise spatial correlations between each vertex's RSFC correlation map with that of every other vertex, resulting in a 32k × 32k matrix. 5) Gradient maps (32k × 32k) were generated by computing the first spatial derivative of RSFC similarity maps, which were then smoothed on the surface with a Gaussian kernel (6mm FWHM). In group-level analysis, 6) the gradient maps were averaged across 100 bootstrap samples for each cohort. 7) Using a watershed algorithm ([Gordon et al. 2016](#_ENREF_1)) to detect borders for the averaged gradient maps, cortical boundaries were identified, creating a 32k × 32k matrix. 8) Parcels were detected using the average cortical boundary map.


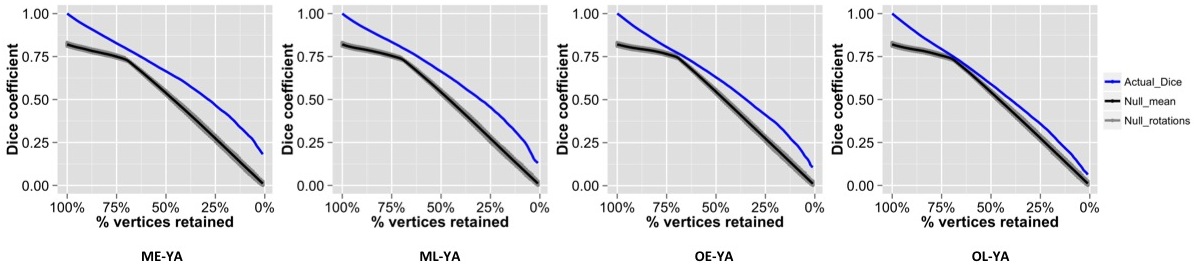


**Figure S2.** **Dice coefficients of boundary maps between YA with each non-YA cohort using Dataset 1**. The Dice coefficients of boundary map similarity between YA and non-YA cohorts (blue line) were compared with the Dice coefficients between randomly rotated boundary maps (null model; black line: mean Dice coefficients of null models; gray shade: the Dice coefficients of random rotations in null models) at multiple thresholds of the boundary maps (top 1% to 100% of possible vertex values, in steps of 1%) for each non-YA and YA cohort pairs. For all cohorts, the Dice coefficients of the actual boundaries were significantly higher than all of their null models across thresholds.

| **Cohorts** | **YA** | **ME** | **ML** | **OE** | **OL** |
| --- | --- | --- | --- | --- | --- |
| **Number of Parcels** | 472 | 469 | 468 | 469 | 522 |
| **Mean Parcel Area (in mm^2^)** | 217.17 | 218.68 | 219.18 | 217.49 | 196.58 |

**Table S1. Number of Parcels and mean parcel area of the parcellations for each of the 5 cohorts using Dataset 1.** The difference in mean parcel surface area between cohorts was not significant (*F*_(4,2395)_ = 2.32, *p* = 0.055).

***Text S1:*** Relationship between head motion residuals and cohort-based parcellation quality.

Studies have revealed that older adults are more prone to head movement ([Van Dijk et al. 2012](#_ENREF_5); [Savalia et al. 2017](#_ENREF_4)) that leads to altered RSFC profiles ([Satterthwaite et al. 2013](#_ENREF_3); [Power et al. 2014](#_ENREF_2)). Even though very strict procedures have been adopted to minimize the source of bias from movement (frame censoring [‘scrubbing’] and global signal regression), the residuals in the retained frames may still contribute to differences in parcel quality evaluations, especially in older adults. To understand whether there exists a relationship between residual head motion and parcellation quality, we first focused on the parcellation quality measures (homogeneity and Silhouette analyses) for the OE subgroup, given that this cohort exhibited the lowest mean homogeneity, *Z*-score of homogeneity, and Silhouette coefficient among all cohorts. Two subgroups of OE participants (Dataset 1; n = 16 in each subgroup) were created using a median split of the frame-by-frame displacement (FD) residuals (i.e., FD in the retained frames following movement processing). The lower-motion subgroup was significantly different from the higher-motion subgroup in terms of their FD residuals (*t*_(30)_ = -7.52, *p* < 0.0001) and number of frames contributing to the analysis (*t*_(30)_ = 4.74, p < 0.0001), there was no significant age difference between the two subgroups (*t*_(30)_ = -1.18, *p* = 0.246).

Applying the OE cohort parcellation (and its random rotations) to each subgroup’s average RSFC maps revealed that the parcellation quality measures were higher in the lower-motion relative to higher-motion OE subgroup (mean homogeneity: 76.44 vs 70.85; *Z*-score of homogeneity: lower-motion subgroup, *Z* = 4.24, *p* < 0.01; vs. higher-motion subgroup, *Z* = 3.10, *p* < 0.01; significant difference in the Silhouette coefficients between the two subgroups: lower-motion > higher-motion: *t*_(82220)_ = 9.13, *p* < 0.0001; Figure S3). This suggests that some of the parcellation quality differences across cohorts may relate to lingering differences in the impact of residual head motion on RSFC data.

To more fully understand whether the age-related differences in parcellation quality were entirely mediated by differences in residual motion, we conducted an additional comparison that directly tested this possibility. A subgroup of YA cohort participants (n = 16) was identified who were matched with OE cohort lower-motion subgroup based on each subject’s FD residuals. The YA FD-matched subgroup was significantly different from OE lower-motion subgroup in terms of age (*t*_(30)_ = -32.33, *p* < 0.0001), but not different in terms of FD residuals (*t*_(30)_ = 0.85, *p* = 0.404), or number of frames contributing to the analysis (*t*_(30)_ = 0.48, *p* = 0.637). Applying each cohort-specific parcellation (and its rotations) to its own subgroup’s data revealed that the parcellation quality measures were still different between these subgroups that had now been equated on residual movement and number of frames contributing to the parcellations (mean homogeneity of YA and OE: 82.43 vs 76.44; *Z*-score of homogeneity: YA FD-matched subgroup, *Z* = 9.96, *p* < 0.01; vs. OE lower-motion subgroup, *Z* = 4.24, *p* < 0.01; significant difference in the Silhouette coefficients between the two subgroups: YA > OE: *t*_(82356)_ = 38.03, *p* < 0.0001; Figure S3). These results suggest that even when controlling for head motion residuals, the younger adult cohorts still exhibits higher parcel quality estimation relative to older adults.

The evidence collectively suggested that the lower parcel quality values in older adults result in part from the residual head motion in the retained frames, but that individual variability in older adults relative to younger population cannot be entirely ruled out.

**
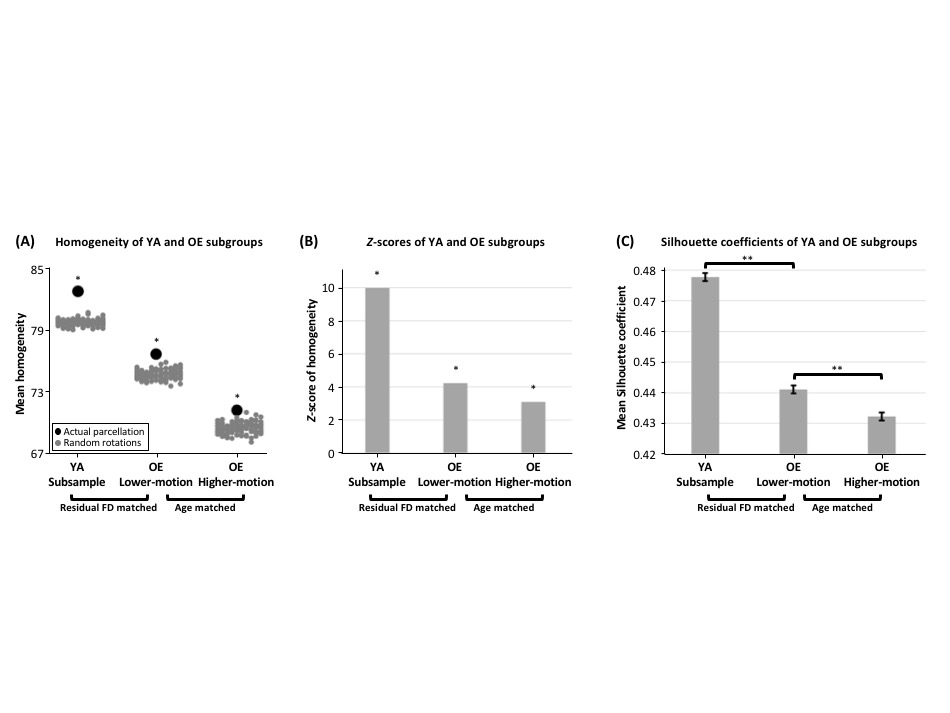
**

**Figure S3**: Residual head motion and differences in data amount relate to part of the differences in parcellation quality across cohorts. Strict steps were taken to account for the influence of head movement on resting-state correlations, including frame-censoring (‘scrubbing’) and global signal regression (see **Methods**). To determine whether the observed differences in parcellation quality across age-cohorts related to any residual head-motion related variance post movement-processing, additional comparisons were made on subgroups of participants from the original cohorts. The OE cohort exhibited the poorest parcellation quality (see **Figure 2** in the main text); this cohort was further divided into a lower-motion OE subgroup and higher-motion OE subgroup based on a median split of the frame-by-frame displacement (FD) residuals (following movement processing). To determine whether motion alone contributed to parcellation quality, a subgroup of YA participants who were matched with OE lower-motion subgroup based on each subject’s FD residuals was also identified and compared. (A) Applying cohort-specific parcels (and rotations) to each subgroup’s data reveals the homogeneity variance across subgroups. For the age-matched OE subgroups, OE lower-motion exhibited a greater difference between homogeneity values using OE-cohort parcels and rotations than OE higher-motion, resulting in better parcel quality (a higher *Z*-score as shown in B). However, the matched YA subgroup exhibited greater difference between homogeneity with YA-cohort parcels and rotations, relative to the OE lower-motion subgroup, demonstrating that residual motion alone does not fully explain the between-cohort parcellation quality differences. (C) Silhouette coefficients were computed and compared for age- and residual FD-matched subgroups; two-sample t-tests parallel the observations in (A) and (B), revealing a significant difference in the mean parcel quality between the age-matched subgroups (OE lower-motion > OE higher-motion) and between residual FD-matched subgroups (YA FD-matched > OE lower-motion). Error bars indicate 95% confidence intervals of mean coefficients. *Significance level for Z-scores of homogeneity and two-sample t-tests of Silhouette coefficient*: **p* =< 0.01; ***p* =< 0.0001.

***Text S2: Description of parcellations derived from the combined dataset***

Functional parcellations were created using each cohort’s data in Dataset 1 combined with the corresponding cohort in Dataset 2.

Similar to the observations made on the parcellations generated only from Dataset 1, visual inspection of the combined dataset's boundary maps reveals that locations of cortical boundaries are similar across all cohorts in some locations, while other RSFC boundary features differ across one or more cohorts' maps (Figure S4A). Direct evaluation of border overlap confirmed that many of the strongest borders were present in each of the 5 independent cohort maps, providing evidence that certain prominent area features do not differ across the adult lifespan (Figure S4C).

The total number of parcels identified in each parcellation map was comparable across cohorts, ranging from 451 to 470. There was no significant difference in mean parcel surface area between cohorts (*F*_(4,2301)_ = 0.2, *p* = 0.939; Table S2).

The combined dataset’s cohort-specific parcellations were applied to their corresponding cohorts’ data and examined with homogeneity tests. Cohort-specific parcels were always more homogenous than their null models (all *Z*s >= 4.58, *p*s < 0.01), as were the YA parcels applied to each cohort (all *Z*s >= 1.94, *p*s =< 0.02). Critically, as observed with the parcellation defined from Dataset 1 alone, cohort-specific parcels exhibited greater homogeneity (Figure S5A) and higher mean Silhouette coefficients (Figure S5B) when applied to their own cohort, relative to YA parcels.


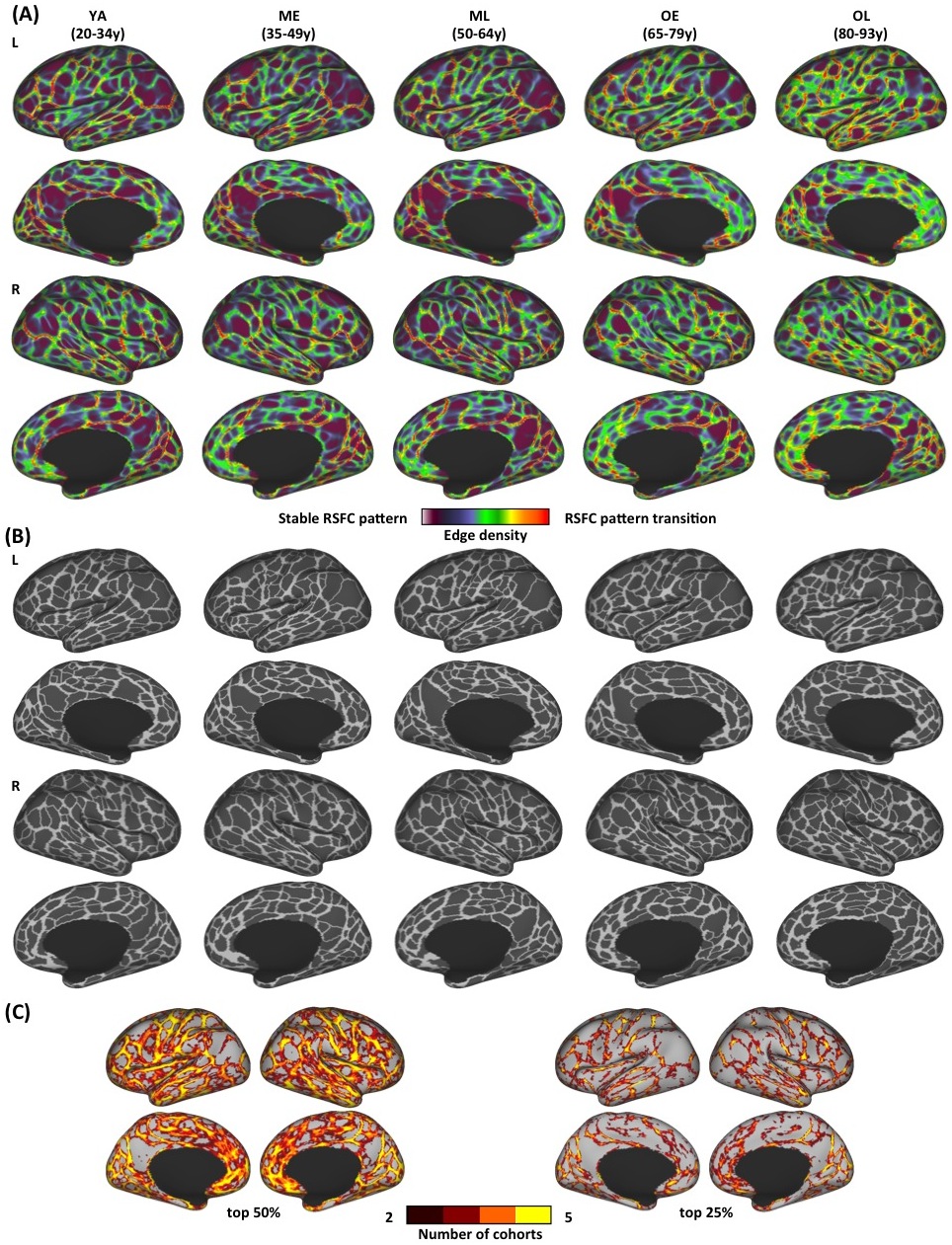


**Figure S4.** **The (A) boundary maps, (B) parcellation maps and (C) boundary conjunction maps using the combined dataset.** (A) RSFC boundary maps of 5 age cohorts: hotter colors indicate higher probability of RSFC pattern transition (i.e. a putative area boundary). Cooler colors correspond to vertices with stable RSFC patterns (i.e. less likely to be area boundaries). (B) Functional parcels of 5 age cohorts, calculated from their corresponding boundary maps. (C) Conjunction images depicting locations where stronger boundaries are consistently identified across cohorts. Each cohort map from (A) was thresholded to its top 50% (left) and 25% (right) boundary map values and summed to reveal boundary features that are consistently identified across at least 2 cohorts. This supplemental figure incorporates additional information to **Figure 3** from the main manuscript.

| **Cohorts** | **YA** | **ME** | **ML** | **OE** | **OL** |
| --- | --- | --- | --- | --- | --- |
| **Number of Parcels** | 470 | 459 | 466 | 451 | 460 |
| **Mean Parcel Area (in vertices)** | 218.17 | 224.01 | 220.33 | 226.81 | 221.57 |

**Table S2.** **Number of parcels and mean parcel area of the parcellations for each of the 5 cohorts using the combined dataset.** There was no significant difference in mean parcel surface area between cohorts (*F*_(4,2301)_ = 0.2, *p* = 0.939).


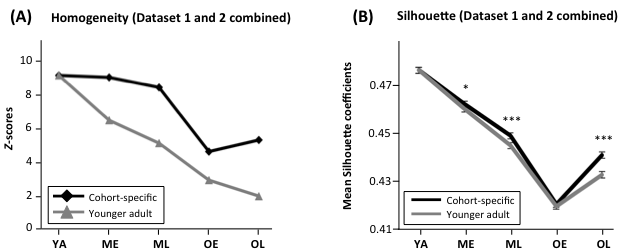


**Figure S5.** **The homogeneity and Silhouette coefficients of the parcellations using the combined dataset.** Cohort-specific functional parcellations of the combined dataset are of high quality and provide a better ‘fit’ to data from their own age range, compared to parcellation defined from younger adults. (A) *Z*-scores of homogeneity values are plotted for cohort-specific parcellations and YA parcellation applied to each cohort’s data. The homogeneity of both cohort-specific and YA parcellations are significantly higher than their corresponding null models, however, cohort-specific parcellations provide a better representation of their corresponding data, as indicated by higher homogeneity *Z*-scores. (B) Except for OE, mean Silhouette coefficients of cohort-specific parcellations are significantly higher than YA parcellation in both datasets, suggesting that in general, cohort-specific parcellations more reliably define brain areas in their corresponding age ranges. Error bars indicate 95% confidence intervals of mean coefficients. *All Z-scores of homogeneity tests*: *p* =< 0.02; *significance levels for two-sample t-tests of Silhouette coefficient*: **p* < 0.05; ****p* < 0.001.


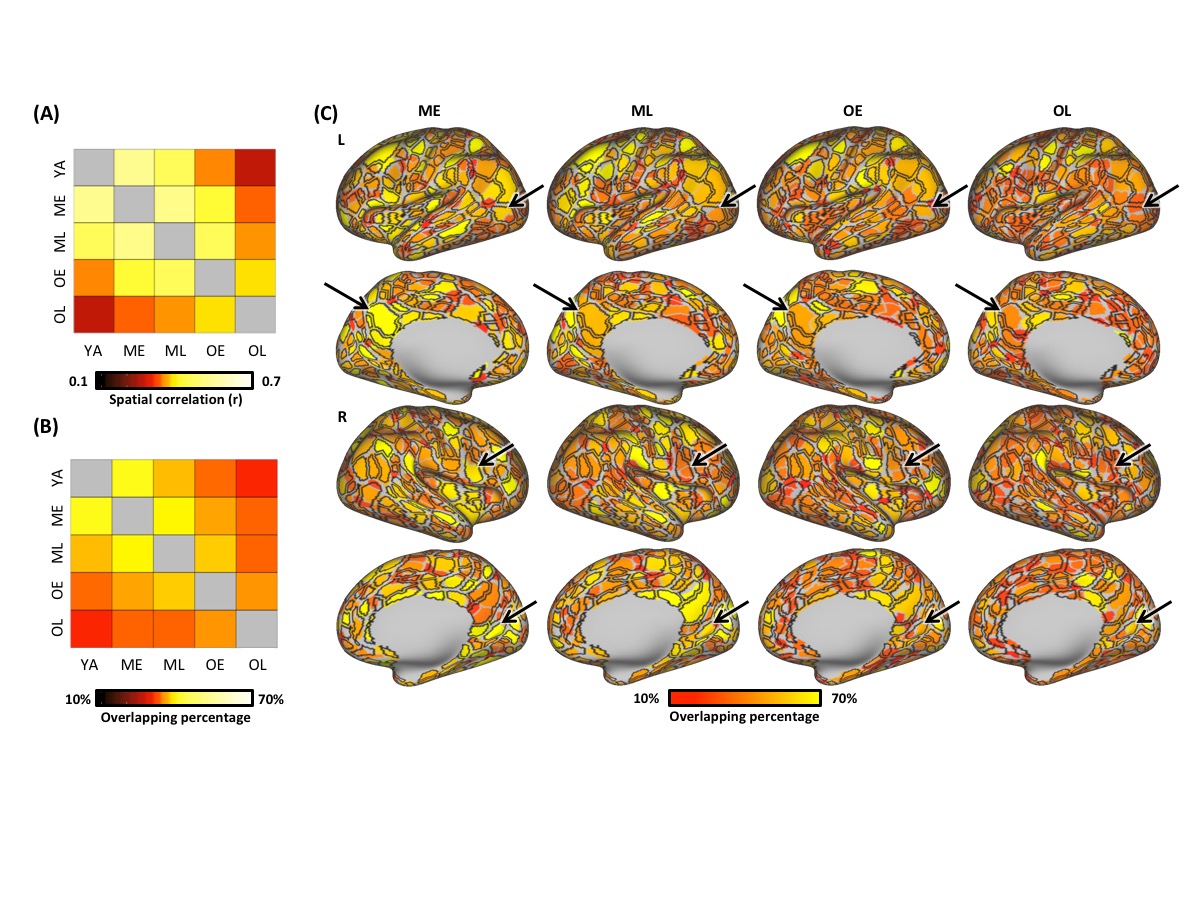


**Figure S6. The spatial similarity of boundary and parcellation maps using the combined dataset.** (A) The boundary map of each cohort is more similar to cohorts more proximal in age (adjacent cells close to diagonal) than cohorts that are more distant in age. Each color square of the plot represents the spatial correlation coefficient (*r*) of the boundary maps between each cohort pair. (B) The similarity of cohorts’ parcellations resemble the trend revealed in (A), with greater parcellation similarity between cohorts that were derived from participants that were closer in age. Each color square of the plot represents the mean spatial overlapping percentage across all matched parcels between any given pair of cohorts. (C) Non-YA parcels exhibit variable spatial correspondence to YA parcels. Using the parcellations derived from the combined datasets, the parcels for each non-YA cohort (underlay) are colored by their values of overlapping percentage with YA parcels (overlaid black borders) that were matched according to maximal spatial overlap. Arrows point to example parcels with relatively high spatial overlap across cohorts, although the % overlap decreases with increasing age.

***Text S3: Relationship between variability of anatomical alignment, cortical thickness differences and boundary differences***

*Variability of anatomical alignment is greater in the oldest cohort*. To evaluate the differences in anatomical alignment across cohorts, a one-way ANOVA was performed on each individual’s mean absolute deformation value across the cortical surface, grouped by their age-cohort. There was a significant main effect of cohort (*F*_(4,354)_ = 12.06, *p* < 0.0001). *Post*-*hoc* comparisons between non-YA and YA deformation values revealed that the OL subjects’ mean deformation values were significantly greater than YA subjects (*t*_(132)_ = 4.59, *p*_(Bonferroni-corrected)_ < 0.0001), suggesting greater degree of anatomical realignment due to individual variability of anatomy for the oldest cohort relative to younger adults.

*Relationship between age-related differences of anatomical alignment, cortical thickness and functional boundaries*. Across vertices, the correlation comparing (1) the difference in cortical thickness between each non-YA cohort and the YA cohort and (2) the deformation difference between the groups was computed to assess the spatial relationship between the two. With the exception of the ME cohort (*r* = 0.006, *p* = 0.146), all 3 cohorts exhibited small but significant correlations (ML: *r* = 0.022, *p* < 0.0001; OE: *r* = 0.009, *p* = 0.022; OL: *r* = 0.044, *p* < 0.0001), suggesting that the cortical locations (vertices) in which there is greater cortical thickness differences exhibit some correspondence to vertices exhibiting deformation differences as well.

Statistical tests were conducted to determine whether differences in cortical thickness or alignment exhibited distinct spatial distributions and magnitudes across vertices with boundary overlaps (YA vs. non-YA cohort) and vertices with no boundary overlaps. Across multiple thresholds (top 50% and top 25% of the boundary map values), $\chi^{2}$ tests revealed no significant relation between the differences in thickness or deformation and the amount of boundary overlaps between the YA cohort and each of the non-YA cohorts (all *p*s > 0.05). However, consistent with the correlations presented earlier, two-sample *t*-tests revealed that when greater boundary map values were retained (top 25% of the valid vertices), the comparison between the thickness of vertices with overlapping boundaries (YA vs. non-YA cohort) to vertices with no overlapping boundaries, the thickness difference was greater in locations where there was no overlap (OE: *t*_(14852)_ = 4.682, *p* < 0.0001; OL: *t*_(14852)_ = 3.079, *p* = 0.002); these differences were absent in middle-age cohorts (ME: *t*_(14852)_ = 0.808, *p* = 0.419; ML: *t*_(14852)_ = 1.728, *p* = 0.084). Similar effects were not found in relation to deformation differences when comparing locations that exhibited boundary overlaps across cohorts (*t*_[ME,ML,OE,OL]_ = [0.122, -2.587, -0.236, -0.44], *p* = [0.903, 0.010, 0.814, 0.660]).


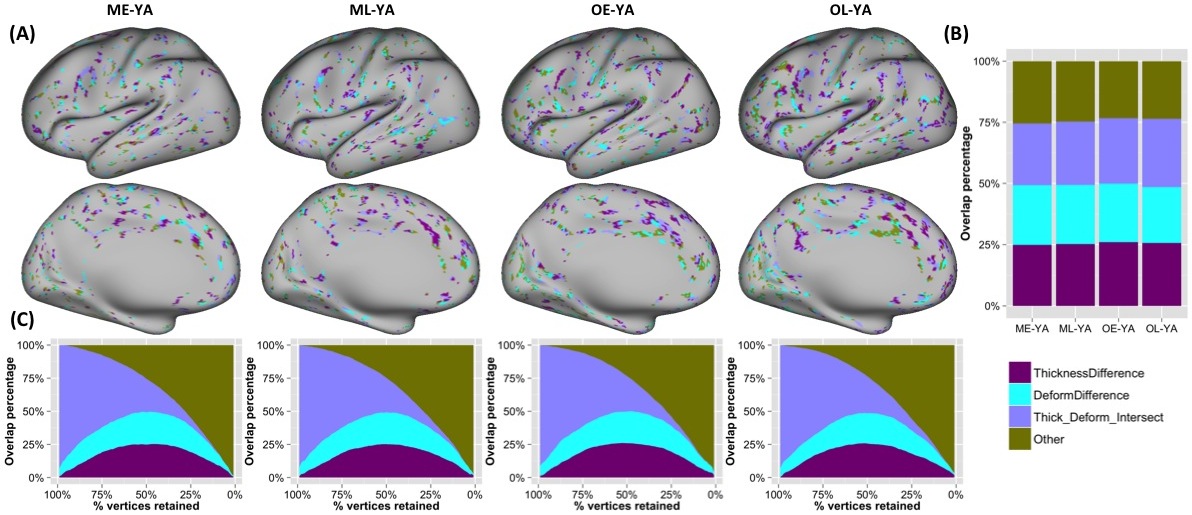


**Figure S7.** **Correspondence between locations exhibiting RSFC-defined boundary differences and anatomical differences.** Locations where a boundary was detected in a non-YA cohort but not the YA cohort were examined (as a compliment to Figure 5). (A) At a threshold of 50%, for the locations where boundaries were present in the non-YA cohorts but absent in the YA cohorts, the majority of vertices are locations that exhibit between-cohort differences in either cortical thickness (purple), anatomical alignment (deformation; cyan), or their overlap (lavender). Vertices colored olive correspond to locations where there exists a boundary in the non-YA map but not the YA map, and that did not exhibit between-cohort differences in either cortical thickness or anatomical alignment. The distribution and proportion of these relationships is plotted in (B). For each cohort’s non-overlap boundary, its major portion (~75%) is at the locations exhibiting differences in the deformation map and cortical thickness maps (all thresholded to retain the top 50% of vertices). (C) When a higher threshold is used (fewer vertices with stronger map values retained), the differences in boundary overlap are more likely to be explained by either anatomical deformation or cortical thickness difference, but not their spatial intersection.


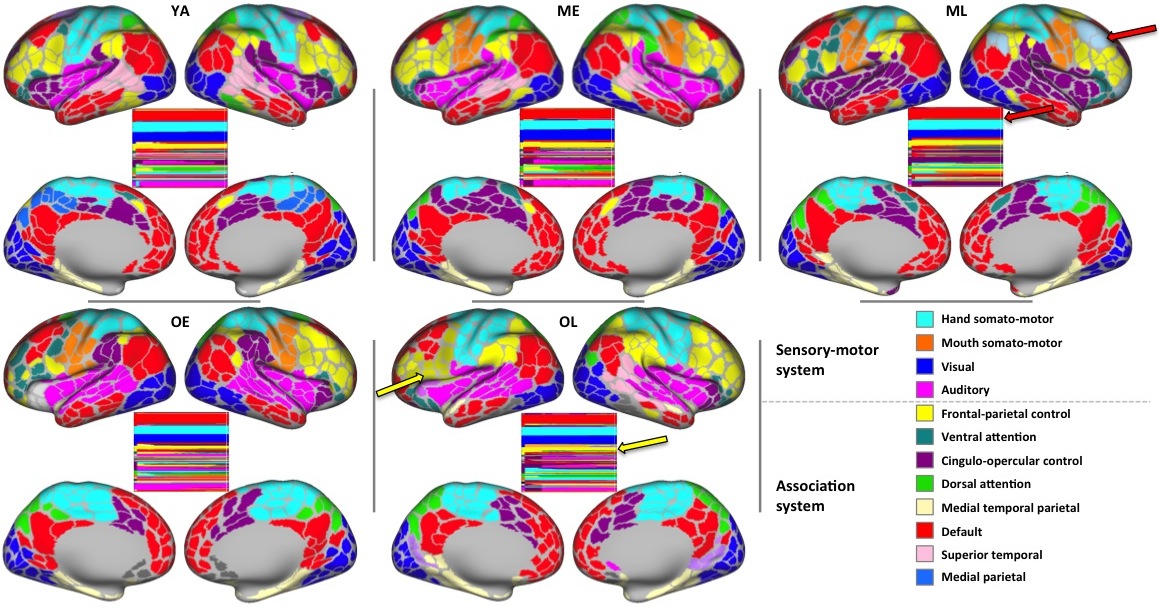


**Figure S8.** **The un-altered community assignments for the 5 cohorts.** Using cohort-specific parcels as nodes, communities were detected based on the resultant network’s times-series across a range of edge-density thresholds (see Main Text for details). As shown in the color bars that reflect the community assignment labels across various thresholds of edge densities (along the x axis, the leftmost edge density is 10% while the rightmost edge density is 3%; nodes (parcels) on the y axis). Our algorithmic assignment procedure summarizes the community assignment across thresholds. In most cases this assignment reflects the overall pattern, but some parcels that are associated with larger communities across most edge density thresholds became isolated into smaller communities at sparser thresholds (see arrows). These parcels were reassigned to the communities they originated from (i.e., was a part of at denser thresholds) to account for this over-partitioning at the restricted edge-density range, here we present the unaltered assignments (reassigned communities shown in Figure 6). For example, in ML, a group of parcels in the left lateral prefrontal cortex (red arrows) has been assigned to the default mode community (system) across a broad range of edge-density thresholds, however they become isolated from this community at higher thresholds; this pattern was not observed in any other age cohort so the nodes were manually reassigned to more accurately reflect their probable community label. Similarly, a collection of nodes broadly assigned to the frontal-parietal community in OL was partitioned at higher edge-density thresholds (yellow arrows) and were thus manually re-assigned to the frontal-parietal community from which they originated for analysis.

**REFERENCES**

Gordon EM, Laumann TO, Adeyemo B, Huckins JF, Kelley WM, Petersen SE. 2016. Generation and evaluation of a cortical area parcellation from resting-state correlations. Cereb Cortex. 26:288-303.

Power JD, Mitra A, Laumann TO, Snyder AZ, Schlaggar BL, Petersen SE. 2014. Methods to detect, characterize, and remove motion artifact in resting state fmri. Neuroimage. 84:320-341.

Satterthwaite TD, Wolf DH, Ruparel K, Erus G, Elliott MA, Eickhoff SB, Gennatas ED, Jackson C, Prabhakaran K, Smith A, Hakonarson H, Verma R, Davatzikos C, Gur RE, Gur RC. 2013. Heterogeneous impact of motion on fundamental patterns of developmental changes in functional connectivity during youth. Neuroimage. 83:45-57.

Savalia NK, Agres PF, Chan MY, Feczko EJ, Kennedy KM, Wig GS. 2017. Motion-related artifacts in structural brain images revealed with independent estimates of in-scanner head motion. Hum Brain Mapp. 38:472-492.

Van Dijk KR, Sabuncu MR, Buckner RL. 2012. The influence of head motion on intrinsic functional connectivity mri. Neuroimage. 59:431-438.
